# Artificial Diploid *Escherichia coli* by a CRISPR Chromosome‐Doubling Technique

**DOI:** 10.1002/advs.202205855

**Published:** 2023-01-15

**Authors:** Pengju Wang, Dongdong Zhao, Ju Li, Junchang Su, Chunzhi Zhang, Siwei Li, Feiyu Fan, Zhubo Dai, Xiaoping Liao, Zhitao Mao, Changhao Bi, Xueli Zhang

**Affiliations:** ^1^ Tianjin Institute of Industrial Biotechnology Chinese Academy of Sciences Tianjin 300308 P. R. China; ^2^ Key Laboratory of Systems Microbial Biotechnology Chinese Academy of Sciences Tianjin 300308 P. R. China; ^3^ College of Life Science Tianjin Normal University Tianjin 300382 P. R. China; ^4^ School of Biological Engineering Dalian Polytechnic University Dalian 116034 P. R. China; ^5^ Biodesign Center Key Laboratory of Systems Microbial Biotechnology Tianjin Institute of Industrial Biotechnology Chinese Academy of Sciences Tianjin 300308 P. R. China

**Keywords:** bio‐evolution, CRISPR, polyploidy, synthetic biology

## Abstract

Synthetic biology has been represented by the creation of artificial life forms at the genomic scale. In this work, a CRISPR‐based chromosome‐doubling technique is designed to first construct an artificial diploid *Escherichia coli* cell. The stable single‐cell diploid *E. coli* is isolated by both maximal dilution plating and flow cytometry, and confirmed with quantitative PCR, fluorescent in situ hybridization, and third‐generation genome sequencing. The diploid *E. coli* has a greatly reduced growth rate and elongated cells at 4–5 µm. It is robust against radiation, and the survival rate after exposure to UV increased 40‐fold relative to WT. As a novel life form, the artificial diploid *E. coli* is an ideal substrate for research fundamental questions in life science concerning polyploidy. And this technique may be applied to other bacteria.

## Graphical abstract

An artificial diploid *Escherichia coli* cell constructed by a CRISPR‐based chromosome‐doubling technique has a greatly reduced growth rate and elongated cell. As a novel life form, the diploid *E. coli* is robust against radiation and an ideal substrate for researching fundamental questions in life science concerning polyploidy. And this technique might be applied to other bacteria.

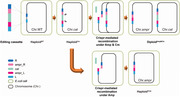

## 1 Introduction

The technological basis of synthetic biology is the construction and implementation of novel genetic systems and remodeling of natural biological systems. This leads to the ultimate question: Can humans create artificial life? In 2010, US scientist J. Craig Venter and his research team reported the world's first semisynthetic life form, a *Mycoplasma* with a nearly identical version of its natural chromosomes synthesized in vitro using chemical methods.^[^ ^1^ ^]^ This work officially heralded the onset of synthetic biology. Since then, the progress of synthetic biology is represented by milestones at the genomic scale, which includes the creation of a bacterium with an entirely synthetic genome^[^ ^1^ ^]^ and the construction of a designer yeast chromosome.^[^ ^2^ ^]^ In 2019, Shao et al. fused the 16 natural chromosomes of the single‐cell eukaryote *Saccharomyces cerevisiae* into a single functional chromosome.^[^ ^3^ ^]^ This work further demonstrated that natural life systems could be significantly modified at the genomic scale by human intervention. These studies demonstrated the great innovation capacity of synthetic biology. Inspired by these great advancements, here we aim to create an artificial polyploid model bacterial strain.

A genome is defined as the haploid (single copy) set of chromosomes with the genes they contain. Cells containing two or more complete sets of chromosomes are designated as diploid or polyploid, respectively. The number of chromosomes is probably the most important property of all organisms, and is genetically conserved. Polyploidy is exceedingly rare in animals, only with a few examples in lower animals, and almost none in higher animals. In the plant kingdom, the situation is quite different, and polyploidy is estimated to be present in half of all plant species. The proportion even increases to two‐thirds in higher plants. Influenced by earlier studies mostly based on model microorganisms such as the *Escherichia coli* (*E. coli*) (Gram‐negative) and *Bacillus subtilis* (Gram‐positive), bacteria are usually considered to only contain one circular chromosome.^[^ ^4^ ^]^ However, with the expansion of study scope and fast development of genome sequencing, polyploid bacteria and archaea were recently discovered, which mainly belong to the phyla Cyanobacteria, *Deinococcus‐Thermus* and *Euryarchaeota*.^[^ ^5^, ^6^, ^7^ ^]^

Biological polyploidy may involve multiple intracellular biological processes, and imbue organisms with growth advantages. Many crops including coffee, bananas, peanuts, tobacco, kiwifruit, and strawberries were unwittingly selected as polyploids for their exaggerated traits such as large fruits, seeds, and leaves.^[^ ^8^ ^]^ In recent years, considerable progress has been made in the study of the mechanisms of eukaryotic polyploidy and genome biology.^[^ ^9^, ^10^, ^11^ ^]^ Since there was only culturing method to obtain corresponding haploid and polyploid plants and yeasts, scientific research concerning the mechanism and function of polyploidy was mainly limited to a few species of eukaryotes.^[^ ^12^, ^13^ ^]^ Due to the lack of molecular technique to create artificial haploid and polyploid cells for the comparison study, research focusing on polyploidy in other kingdoms were very rare. A simple research model, ideally a fundamental model cell with both haploid and diploid configuration is necessary to be constructed for the polyploidy studying.

With the development of CRISPR genome editing techniques, researchers have obtained much more powerful tools to manipulate cellular chromosomes than before. In this work, we developed a theoretically universal CRISPR‐based technique to create artificial polyploid bacteria. We successfully created the first model organism, *E. coli*, to contain two chromosomes. This work can provide an ideal pair of objects, diploid and haploid *E. coli*, for conducting experiments for answering quite a few fundamental biological questions concerning the polyploidy.

## 2 Results

### 2.1 Design of a CRISPR‐Based Chromosome‐Doubling Technique

**Figure 1:**
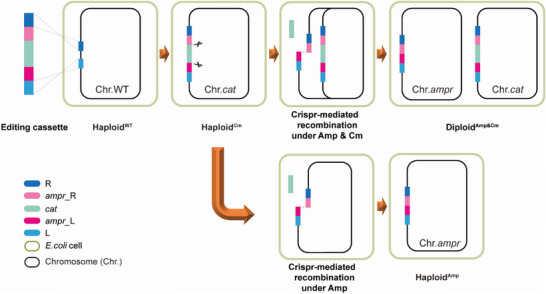
Schematic of the CRISPR‐based chromosome‐doubling technique. The editing cassette is inserted into the chromosome by homologous recombination, forming a chromosome bearing a chloramphenicol resistant gene (Chr.*cat*). Subsequently, the CRISPR/Cas9 system is expressed to generate two double‐strand breaks (DSB) at two inserted N20PAM sequences, after which a DSB‐mediated intra‐chromosomal recombination event between the *ampr*_L and *ampr*_R homologous arms forms a second chromosome carrying a complete *ampr* gene (Chr.*ampr*). When both ampicillin and chloramphenicol are added, a Diploid^Amp&Cm^ *E. coli* is generated. As a control, Haploid^Amp^ *E. coli* containing the *ampr* gene bearing chromosome (Chr.*ampr*) is generated when only ampicillin is added.

In order to create a second chromosome in *E. coli*, a CRISPR‐based strategy was designed as illustrated in **Figure** **1**. First, we inserted an editing cassette carrying the functional elements, including a left homologous arm (L), the left half of the ampicillin resistance gene (*ampr*_L), two CRISPR/Cas9 recognition regions (N20PAM), a chloramphenicol resistance gene (*cat*), the right half of ampicillin resistance gene (*ampr*_R), and a right homologous arm (R), into a target locus on the *E. coli* chromosome by homologous recombination, forming a chromosome bearing a chloramphenicol resistance gene (Chr.*cat*). Subsequently, a CRISPR/Cas9 system was expressed with gRNA to induce two double‐strand breaks (DSB) at two inserted N20PAM sequences inserted as part of the editing cassette, which caused a DSB‐mediated intra‐chromosomal recombination event between the *ampr*_L and *ampr*_R homologous arms. Thus, a second chromosome carrying an ampicillin resistant gene (Chr.*ampr*) was created from the original chromosome (Chr.*cat*). When both ampicillin and chloramphenicol were supplemented, the antibiotic pressure forced the cell to retain both chromosomes, and a diploid *E. coli* was created (Diploid^Amp&Cm^). As a control, Haploid^Amp^ *E. coli* containing Chr.*ampr* was generated when ampicillin was supplemented. We designed the method employing fundamental cellular mechanisms, so that it might be universally applicable to prokaryotic organisms.

### 2.2 Confirmation of the Diploid *Escherichia coli* Created Using the CRISPR‐Based Chromosome‐Doubling Technique

Starting from the wild‐type *E. coli* strain MG1655, designated as Haploid^WT^, the editing cassette was integrated into the chromosome between the *ddp*X and *dos*P genes to obtain the strain Haploid^Cm^. The timing of inducing the cleavage by Cas9 is the key to the successful preparation of diploid *E. coli*. The Haploid^Cm^ *E. coli* was cultured with fast growing medium. During the exponential phase, multiple replication origins, termini and editing cassettes were presented (Figure 1). At that time, the CRISPR/Cas9 system was induced to make the DSB at one editing cassette, which caused a DSB‐mediated intra‐chromosomal recombination event. Thus, a second chromosome carrying an ampicillin resistance gene, Chr.*ampr*, was formed in the same cell along with the Chr.*cat* chromosome, resulting in the Diploid^Amp&Cm^ *E. coli*. To prevent two copies from being cut by the Cas9, the induction strength was also critical, which could not be too strong that all chromosomes were cut. At least a few cells were allowed to maintain the Chr*.cat* and survive. In fact, most *E. coli* cells were killed at the process, that after overnight induction, a large number of dead cells appeared. The timing of ampicillin addition and inoculation ratio were also important. After overnight induction, cells were transferred into fresh LB medium with ampicillin and the other three antibiotics at an inoculation ratio of 30%. After the culture recovered growth, it was spread on LB agar plates with ampicillin, kanamycin, apramycin, and chloramphenicol.

**Figure 2:**
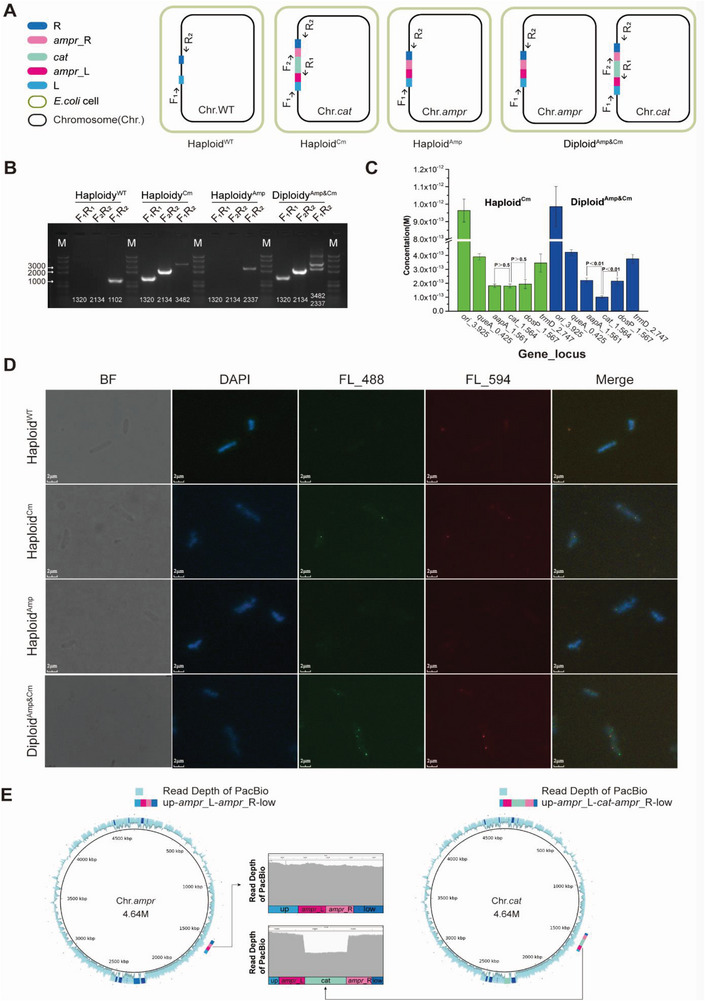
Confirmation of Diploid^Amp&Cm^ *E. coli* with colony PCR, qPCR, FISH, and genome de novo sequencing. A) Three primer pairs were used to amplify the diagnostic bands spanning the *ampr* and *cat* regions. The primer position in the four *E. coli* variants is indicated in the figure. B) Gel picture of colony PCR products with the genomic DNA of the four *E. coli* variants as template. C) Histogram of the concentrations of six genes in Haploid^Cm^ and Diploid^Amp&Cm^ strains adopted from Real‐time PCR. D) The *ampr*_L–*ampr*_R and *ampr*_L–*cat*–*ampr*_R configurations in four *E. coli* variants were visualized by two‐color FISH, the nucleoids were visualized by DAPI staining with fluorescent blue color. The a*mpr*_L–*ampr*_R configuration was probed by the *ampr* probe with fluorescent green color, while a*mpr*_L–*cat*–*ampr*_R configuration was probed simultaneously by the *ampr* probe and the *cat* probe, and appeared fluorescent yellow color, a mixture of fluorescent green color and fluorescent red color. E) Characterization of the Diploid^Amp&Cm^ *E. coli* genome, and Circos plot of the Chr.*ampr* and Chr.*cat*.

To identity the diploid *E. coli* strain, a single colony was picked to ensure the purity of the population, which served as the template of identification PCR. Three primer pairs were designed to amplify the key regions spanning the *ampr* and *cat* cassettes, which were specifically present in Chr.*cat* and Chr.*ampr*, respectively (**Figure** **2**A). For the wild‐type strain Haploid^WT^, only the primers F_1_ and R_2_, both with DNA sequences from the chromosome, could generate an amplification product with a size of 1101 bp (Figure 2B). After integrating the editing cassette, amplification products could be obtained from Haploid^Cm^ *E. coli* with all three primer pairs, including F_1_R_2_^Cm^ (3482 bp), F_1_R_1_ (1320 bp), and F_2_R_2_ (2134 bp). After the recombination event under ampicillin selection pressure, Haploid^Amp^ *E. coli* containing the Chr.*ampr* only yielded a specific PCR band with the F_1_R_2_ ^Amp^ primer pair, giving a product of 2337 bp. For identification of the diploid strain containing both Chr.*ampr* and Chr.*cat*, PCR products were obtained with all three primer pairs as illustrated in Figure 2B. Especially with primer pair F_1_R_2_, two amplification products were obtained, one with a size of 2337 bp (F_1_R_2_^Amp^) amplified from Chr.*ampr*, and one with a size of 3482 bp (F_1_R_2_^Cm^) amplified from Chr.*cat*. The PCR identification experiment proved that the diploid *E. coli* strain was successfully constructed.

In order to confirm the single‐cell status of the tested *E. coli* cells, we experimented with more methods to ensure that the tested population was uniformly grown from single‐cell, but not the mixture of more than one strain. First, we adopted the maximal dilution and plating method to obtain single‐cell formed colonies on the LB agar plate (Figure S1, Supporting Information). Second, we used the flow cytometry method to separate and sort out single cells (Figure S2, Supporting Information). Then we used the single‐cell formed cell culture for analyzing the chromosomal configuration. Specifically, for the maximal diluting and plating method, we initially used the microscope to ensure that the *E. coli* cells in the culture were uniformly dispersed without adhesion (Figure S1A, Supporting Information). Next, the culture was further diluted by 10^4^‐ and 10^6^‐fold, and subjected to the flow cytometry sorting and maximal dilution plating, respectively. With the maximal diluting method, well separated colonies were dispersedly located on the agar plates, which indicated the single‐cell status was achieved. The basic function of the flow cytometry technique is to efficiently sort single particles with the sizes from 0.5 to 40 µm. With the hydrodynamic focusing mechanisms, the single‐cell suspension was ejected as single‐cell lines from the fluid chamber of flow cytometer, and formed single‐cell colonies on the agar plates. To identity the diploid *E. coli* strain, a group of 32 single colonies and a group of 24 single colonies were picked for PCR analysis. Three pairs of primers were used for the validation and the obtained PCR bands showed that all 56 single colonies had the genome configuration of diploid *E. coli* (Figures S1C and S2C, Supporting Information).

### 2.3 Confirmation of Diploid *Escherichia coli* by Quantitative PCR

Since the diploid *E. coli* underwent a complete chromosomal duplication, only the *cat* gene was present as a single copy, while the *ampr* gene and all the endogenous DNA sequences were doubled, even though *ampr* was split by *cat* into *ampr*_L and *ampr*_R on Chr.*cat*. To confirm this, we used a quantitative PCR method adapted from Breuert et al.^[^ ^14^ ^]^ to determine the copy number of the *cat* gene and five endogenous genes. The selected genes and loci were *ori*C_3.925, *que*A_0.425, *ddp*A_1.561, *cat*_1.564, *dos*P_1.567, and *trm*D_2.747. The *ori*C gene was from replication origin, and the *ddp*A gene and *dos*P gene were adjacent to the *cat* gene. While *que*A gene was located between *ori*C gene and *ddp*A gene, and *trm*D gene was between *ori*C gene and *dos*P gene. And their relative loci on the Haplid^Cm^ *E. coli* genome physical map are shown in Figure S3A, Supporting Information.

A fragment of ≈1 kbp was amplified in the *cat* gene, and five endogenous genes were used as references. A series of dilutions of the six samples were prepared, quantified spectrophotometrically, and used as standards for quantitative PCR. The length of the six qPCR fragments was designed to range from 105 to 115 bp (Table S6, Supporting Information). To obtain a standard curve, the logarithm of the molar concentration of the standard sample was plotted to the abscissa, and the measured Ct value was plotted to the ordinate (Figure S3B, Supporting Information). Thus, the molar concentration of a given sample of *cat* and five endogenous genes could be determined from the standard curve according to its Ct. The molar concentration of the *ampr* gene was not measured because no appropriate qPCR primer pairs could be designed.

In the control Haploid^Cm^ *E. coli* strain, the molar concentration of the *cat* gene was determined to be 1.81 ± 0.14 × 10^−13^ m, while those of *ddp*A and *dos*P, adjacent to the editing cassette, were determined to be 1.82 ± 0.12 × 10^−13^ m and 1.94 ± 0.32 × 10^−13^ m in the same sample, there was no significant difference between them (*p* > 0.5). The molar concentration of the *ori*C gene was determined to be 9.63 ± 0.66 × 10^−13^ m, much higher than that of the *cat* gene, adjacent to terminus. Similarly, the molar concentration of *que*A was 3.89 ± 0.23 × 10^−13^ m between *ori*C and *ddp*A, and *dos*P was 3.89 ± 0.23 × 10^−13^ m between *ori*C and *dos*P. These results suggested that the *cat*, *ddp*A, and *dos*P genes had the same copy number in the Haploid^Cm^ *E. coli* strain, which were lower than the amount of other three endogenous genes close to *ori*C. Using a sample prepared from the diploid *E. coli*, the molar amount of the *cat* gene was determined to be 1.01 ± 0.09 × 10^−19^ M, while that of *ddp*A and *dos*P, adjacent to the *cat* gene, were determined to be 2.22 ± 0.18 × 10^−13^ M and 2.16 ± 0.24 × 10^−13^ M. The molar amount of the endogenous gene *ddp*A and *dos*P were roughly twofold that of the *cat* gene, respectively, and the difference was significant (*p* < 0.01), which confirmed the diploid status of the analyzed *E. coli* strain. Similarly, the amount of other three endogenous genes adjacent to the *ori*C were higher than that of *cat* gene close to terminus, which were 4.22 ± 0.20 × 10^−13^ m for *que*A, 3.75 ± 0.31 × 10^−13^ m for *trm*D, and 9.86 ± 1.14 × 10^−13^ m for *ori*C respectively (Figure 2C and Figure S3C, Supporting Information).

### 2.4 Confirmation of Diploid *Escherichia coli* by Fluorescent In Situ Hybridization

Since Chr.*cat* and Chr.*ampr* in the diploid *E. coli* were almost identical, except that the Chr.*cat* contains an a*mpr*_L–*cat*–*ampr*_R configuration, while Chr.*ampr* contains an a*mpr*_L–*ampr*_R configuration. The *ampr* and *cat* genes of Diploid^Amp&Cm^ *E. coli* were probed simultaneously by two‐color fluorescence in situ hybridization (FISH). The probe of *ampr* was stained by Alexa Fluor 488 dye, and the probe of *cat* was stained by Alexa Fluor 594 dye. In the Hapliod^WT^ *E. coli* cells, only the Chr.WT stained with DAPI dye with fluorescent blue color could be observed. In the Haploid^Cm^ *E. coli* cells, in addition to Chr.*cat* stained with DAPI dye with fluorescent blue color, the *ampr*_L–*cat*–*ampr*_R configuration could also be observed, which was defined by the *cat* probe based on the *cat* gene, and defined by the *ampr* probe based on *ampr*_L and *ampr*_R. However, the three probed loci were almost overlapped, the a*mpr*_L–*cat*–*ampr*_R configuration appeared fluorescent yellow color, a mixture of fluorescent green color and fluorescent red color. In the Haploid^Amp^ *E. coli* cells, in addition to Chr.*ampr* stained with DAPI dye with fluorescent blue color, only the *ampr* gene locus defined by *ampr* probe with fluorescent green color could be observed. In the Diploid^Amp&Cm^ *E. coli* cells, the Chr.*ampr* and Chr.*cat* were stained with DAPI dye with fluorescent blue color. The a*mpr*_L–*ampr*_R configuration locus on the Chr.*ampr* was defined by *ampr* probe with fluorescent green color, and the a*mpr*_L–*cat*–*ampr*_R configuration locus on the Chr.*cat* was defined by *cat* probe and *ampr* probe and appeared fluorescent yellow color, a mixture of fluorescent green color and fluorescent red color. The *ampr*_L–*ampr*_R and *ampr*_L–*cat*–*ampr*_R configurations were observed at different locations within the same cell, which confirmed the diploid status of the analyzed *E. coli* strain (Figure 2D).

### 2.5 De Novo Genome Sequencing of the Diploid *Escherichia coli*

De novo genome sequencing of the diploid *E. coli* was performed using PacBio platform and 191 276 high quality reads with 13 151 bp mean read length were obtained, ≈546.8‐fold coverage. Two polished contigs were assembled using single‐molecule real‐time (SMRT) Link v5.0.1 software, which consist of sequences of Chr.*ampr* and Chr.*cat* (without SNP or indel). Since the sequences of the two chromosomes are almost identical except for an ampicillin resistant gene on the Chr.*ampr* and a chloramphenicol resistance gene on the Chr.*cat*, the sequencing depth of *ampr* and *cat* gene was used to estimate the sequencing depth of Chr.*ampr* and Chr.*cat*, respectively. In addition, we calculated the average sequencing depths of other regions of the two assembled genomes other than *ampr* and *cat* gene. The results showed that the ratio of sequencing depth of *cat* gene, *ampr* gene, and other sequences was 318.8:510.3:546.8, which is consistent with the expected 1:2:2 ratio of them in the diploid *E. coli* (Figure 2E). Therefore, our de novo genome sequencing of the diploid *E. coli* confirmed the diploid configuration.

### 2.6 Diploid Chromosomal Stability of the Diploid *Escherichia coli*

**Figure 3:**
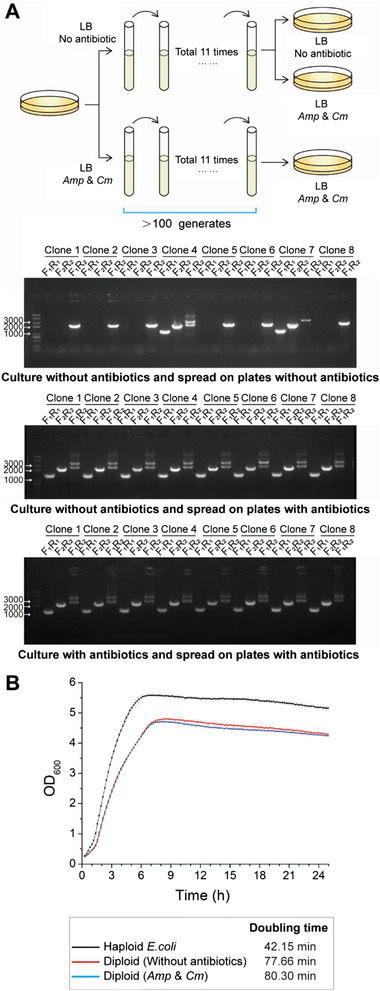
The diploid chromosomal stability and growth status of the diploid *E. coli*. A) Colony PCR analysis of the diploid *E. coli* cells after culturing for more than 100 generations. The amplification of both the 2337 and 3482 bp bands with the primer pair F_1_R_2_ indicates the presence of both Chr.*ampr* and Chr.*cat* in the cell. B) Growth of diploid *E. coli*. Doubling times were calculated from the growth curve data.

The stability of the diploid chromosome configuration is an important property of the artificial diploid *E. coli* we constructed. To study the stability of the diploid *E. coli* population, it was transferred in batch culture for more than 100 generations with or without antibiotics, after which single colonies were subjected to PCR identification as described above (**Figure** **3**A). When the culture and the plate weren't supplemented with antibiotics, only one out of eight tested colonies maintained the diploid chromosomal configuration. Among the remaining haploid colonies, six contained only Chr.*ampr*, and one contained Chr.*cat*. By contrast, when the plate or culture was supplemented with both antibiotics, all eight tested colonies from the two groups maintained the diploid configuration. These results suggested that the diploid *E. coli* chromosomes were highly stable under antibiotic selection. They were less stable without antibiotics, tending to lose either one chromosome to become haploid at a slow rate.

### 2.7 Growth Status of the Diploid *Escherichia coli*

The unnatural chromosomal configuration might have an impact on the proliferation and growth status of the diploid *E. coli*. To analyze the growth status of the diploid *E. coli*, the growth rate of the *E. coli* variants was measured during their exponential phase.^[^ ^15^, ^16^ ^]^ The growth curves were obtained, and the doubling times (DTs) were calculated (Figure 3B). The DT of Haploid^WT^ *E. coli* was 42.15 min, while the DT of Diploid^Amp&Cm^ *E. coli* was 77.66 min without antibiotics and 80.30 min with antibiotics in the culturing system. The DT of Diploid^Amp&Cm^ *E. coli* with/out antibiotic were both proximately twice longer relative to that of the WT, indicating that the antibiotics had a minimal impact on it. To study the stability of genome configuration, two groups of 16 single colonies of the diploid *E. coli* cultured with/out antibiotics were picked for PCR analysis. Three pairs of primers were used for the validation, and the obtained PCR bands indicated that all 32 single colonies remained the genome configuration of diploid *E. coli* (Figure S4, Supporting Information).

### 2.8 Morphological Characteristics of the Diploid *Escherichia coli*

**Figure 4:**
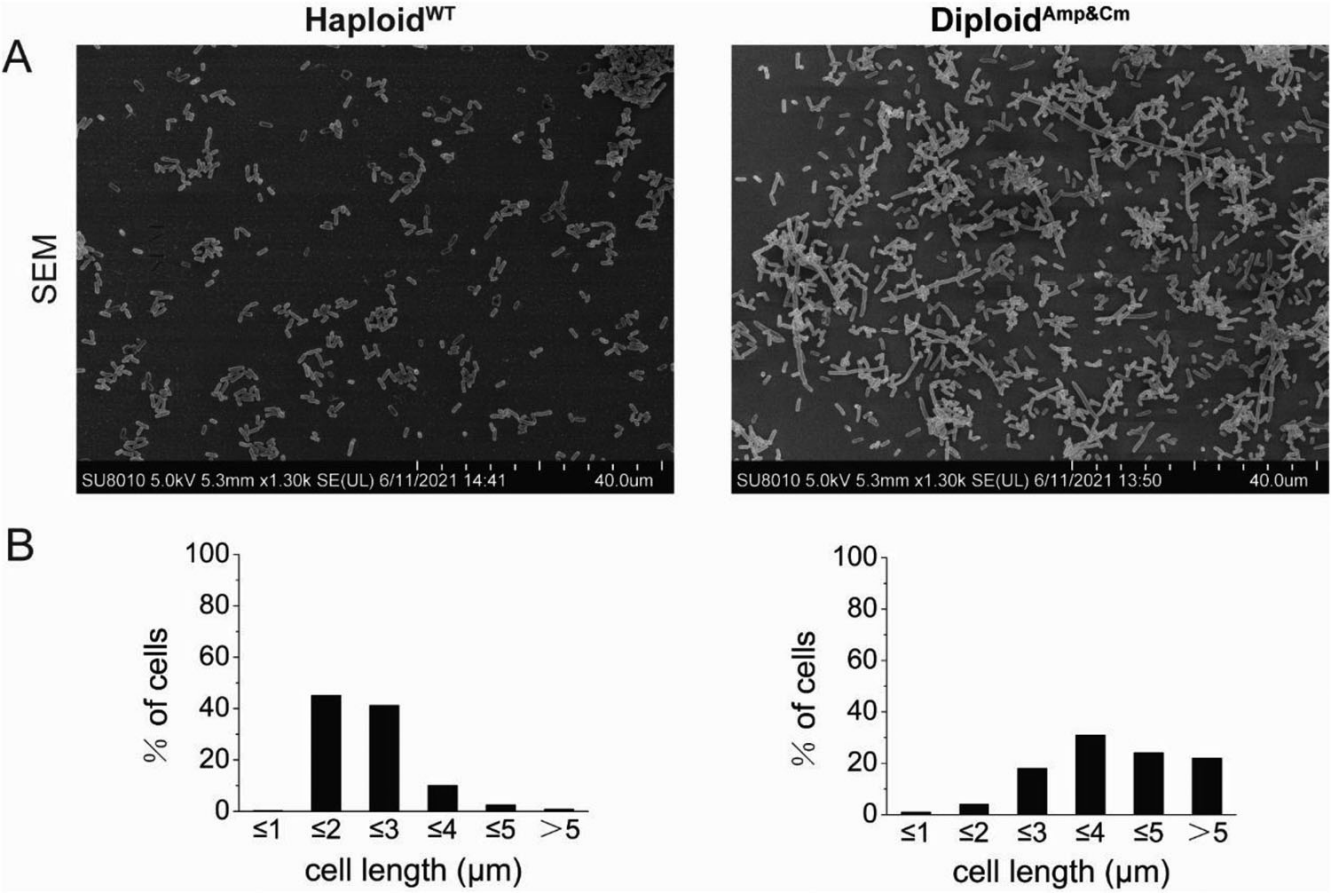
Morphological characteristics of diploid *E. coli*. A) Scanning electron microscopy (SEM) of haploid and diploid *E. coli*. B) Length distribution of haploid and diploid *E. coli*.

Since diploid *E. coli* contains two chromosomes in one cell, we considered that its morphological characteristics might be affected by this significant change. To answer this question, scanning electron microscopy (SEM) was employed to study the morphological characteristics of the diploid *E. coli* in the exponential phase. Compared with the WT, the cells of the diploid *E. coli* cells were obviously elongated, while the control haploid *E. coli* exhibited the typical rod‐shape (**Figure** **4**A). In a visual field containing a few hundred cells, the length of haploid *E. coli* in the exponential phase was between 2 and 3 µm, while the length of diploid cells was mainly between 4 and 5 µm, with some cells being even longer, suggesting that the two‐chromosomal configuration actually enlarged the *E. coli* cells (Figure 4B). It is possible that the diploid status might have affected the proliferation signaling pathways, which increased the DT and delayed the daughter‐cell separation process.

### 2.9 Whole Transcriptome Sequencing of the Diploid *Escherichia coli*

**Figure 5:**
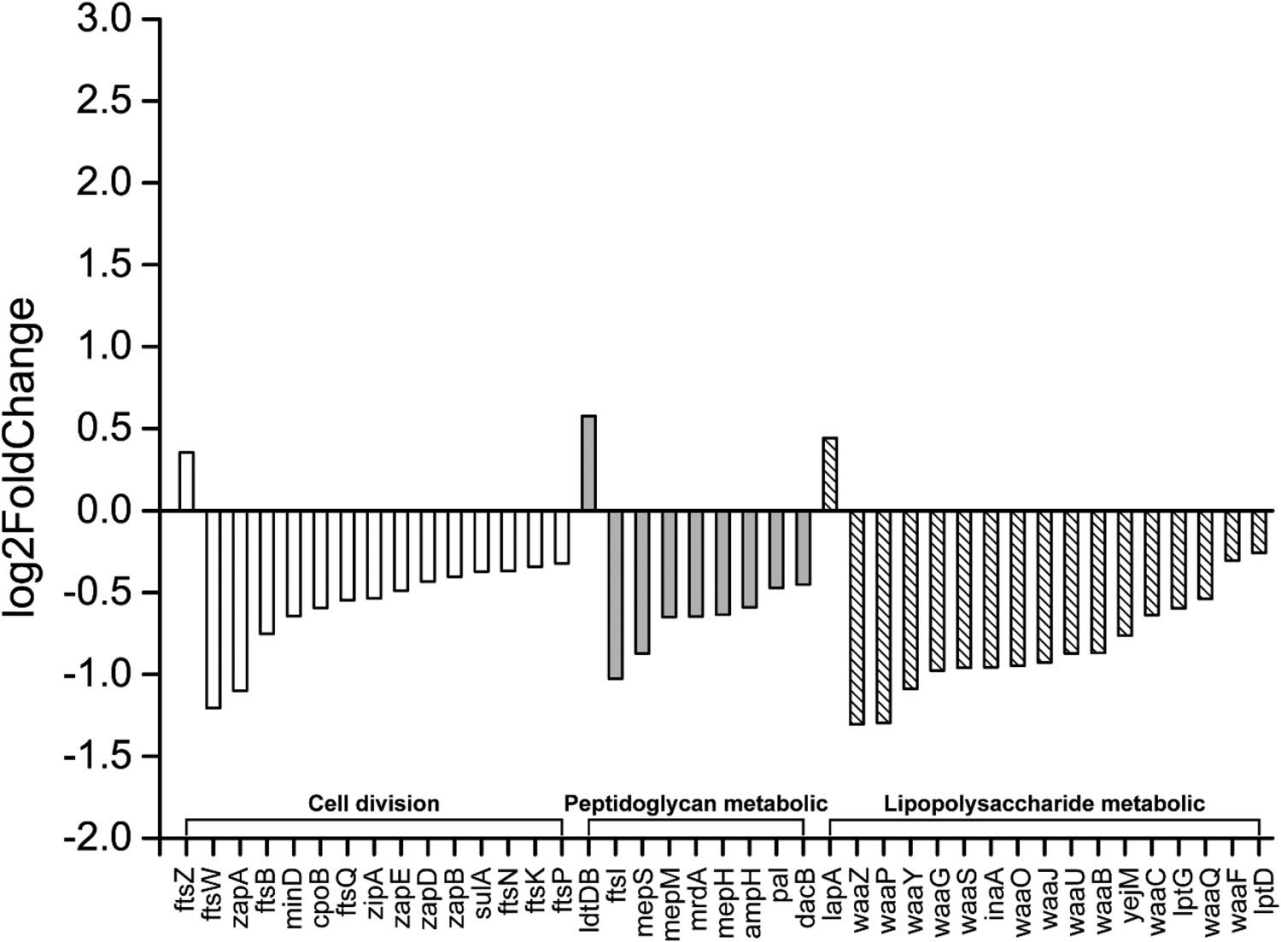
Whole transcriptome RNA‐seq profile of diploid *E. coli*. 15 genes involved in cell division are represented by blank pattern; 9 genes involved in peptidoglycan metabolic are represented by gray pattern; 17 genes involved in lipopolysaccharide metabolic are represented by diagonal pattern.

As mentioned, the diploid status might have affected the proliferation signaling pathways, which increased the DT and delayed the daughter‐cell separation process. To verify this hypothesis, whole transcriptome sequencing (RNA‐seq) was conducted to quantify gene expression of the diploid *E. coli* in the exponential phase. Three independent biological replicates were chosen for haploid and diploid *E. coli*, and each strand‐specific RNA‐seq library was sequenced using an Illumina NovaSeq 6000 system. As shown in the **Figure** **5**, compared to Haploid^WT^ *E. coli*, the expression of 15 genes involved in cell division were significantly reduced except *fts*Z in diploid *E. coli* (*p*‐value = 0.011). In addition, the vast majority of transcription of genes involved in cell wall synthesis was inhibited. For example, the expression of 17 genes involved in lipopolysaccharide metabolic were significantly reduced except *lap*A (p‐value = 0.020), and the expression of 9 genes involved in peptidoglycan metabolic were significantly reduced except *ldt*D (*p*‐value = 0.001). The change in transcriptome was consistent with the cellular growth morphological changes we observed, that the DT was elongated and the cells were enlarged for the diploid *E. coli*.

### 2.10 Survival Rate of Diploid *Escherichia coli* under UV Radiation

Numerous studies investigated the advantages and associated phylogenetic evolution of diploidy and polyploidy. It is commonly believed that the most prominent properties of polyploidy are the ability to survive under extreme environments,^[^ ^17^, ^18^, ^19^ ^]^ probably due to the more efficient repair for DNA breaks,^[^ ^20^, ^21^, ^22^ ^]^ or simply the second and additional chromosomes serving as backups. To our knowledge, there was no direct research on a simple model organism to prove such basic hypotheses. With the artificial diploid *E. coli*, we now have an ideal pair of objects, diploid and haploid *E. coli*, for the fundamental biological experiment.

**Figure 6:**
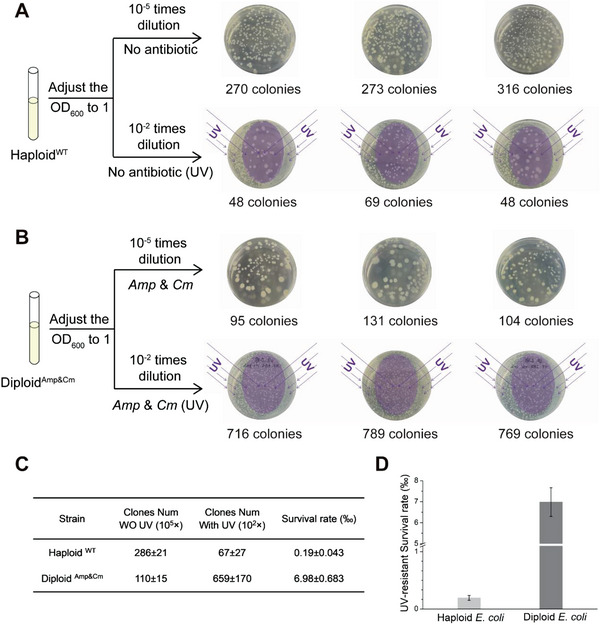
The UV resistance of diploid *E. coli* in comparison with haploid *E. coli*. (A) (B) Diploid and wild‐type haploid *E. coli* cells in the exponential phase were diluted and spread on LB plates with and without antibiotics. The cells were diluted by 10^2^ fold for exposing to UV radiation in a sterile cabinet, and the reference without UV exposure was diluted 10^5^ fold.Since part of the UV radiation was blocked by the side walls of the plastic plate, the colony numbers were counted in an oval shape region of the LB plates.(C) (D) Survival rate of diploid and wild‐type haploid *E. coli* under UV radiation.

Equal numbers of haploid and diploid *E. coli* cells in the exponential phase were plated onto LB agar plates with or without antibiotics, after which the plates were exposed to UV radiation in a sterile cabinet for 4 min, and the surviving cells were allowed to grow into colonies to calculate the survival rate (SR). As illustrated in **Figure** **6**, we mainly counted the colonies in an oval region of the LB plates, since a portion of the UV radiation was blocked by the side walls of the plastic plate. Compared with the control plates with no UV exposure, the populations of both *E. coli* strains were greatly reduced. However, the diploid *E. coli* was found to have a much higher SR than that of the haploid. The haploid *E. coli* had a SR of 0.19 ± 0.043‰ under UV, while that of the diploid *E. coli* was 6.98 ± 0.683‰, which was nearly a 40‐fold increase relative to haploid *E. coli*. Thus, it was experimentally demonstrated for the first time that an artificial diploid form of an organism has a much greater ability to survive under extreme environments, such as UV radiation, than the natural haploid form.

## 3 Conclusion

In this work, we designed a CRISPR‐based chromosome‐doubling method to first construct an artificial diploid *E. coli*. Considering the simple and fundamental mechanism, along with the convenient procedure, we believe that this method can be applied to other prokaryotic organisms. Various analyses confirmed the diploid chromosomal configuration of the constructed strain. The diploid *E. coli* chromosomes were highly stable under antibiotic pressure, and less stable without antibiotics, whereby the cells tended to lose one of the chromosomes to become haploid at a slow rate. As expected, we found that the diploid *E. coli* had a greatly reduced growth rate, which was only half that of the haploid. SEM revealed that most of the diploid *E. coli* cells were obviously elongated to 4 and 5 µm, with a minority being even longer.

Unlike in the eukaryotes, bacterial nucleoids segregation into the daughter cells is achieved without involving an obvious cellular ultrastructure, such as a mitotic spindle. Cell division in *E. coli* is a complex process that requires temporal and spatial coordination of multiple genes. Many *fts* genes involved in cell wall biosynthesis and cell division have been shown to be essential for cell division and cell viability, such as *fts*Z, *fts*Q, *fts*W, and *fts*N.^[^ ^23^ ^]^ Nevertheless, the core proteins are not sufficient for cytokinesis. FtsZ is an essential protein for cell division and cytoskeletal integrity in most bacteria. In *E. coli*, the ratio between FtsZ and FtsA molecules (5:1) is important for proper cell division. It was shown that a two‐ to seven‐fold increase in FtsZ level results in mini‐cell formation, due to additional division events, whereas further higher levels or lower than normal levels cause filamentation due to imbalance in the FtsZ:FtsA stoichiometry.^[^ ^24^ ^]^ Faithful nucleoid segregation, therefore, depends on a precise mechanism that ensures the equipartition of nucleoids among daughter cells as well as the coordination between DNA replication and cell division.^[^ ^25^ ^]^ The best‐understood steps in the process of nucleoid segregation have proven to be the early ones that are required for decatenation and dimer resolution. The subsequent steps of faithful nucleoid partitioning have proven to be more difficult to study, to the point that it has been difficult to postulate even moderately detailed models.^[^ ^26^ ^]^ Since the two chromosomes of the diploid *E. coli* are both complement with the segregation related functional parts, we think that the diploid *E. coli* might undergo a similar segregation process like the wild type. Of course, since there were no cellular mechanisms to ensure faithful distribution of one Chr.*ampr* and one Chr.*cmr* into the daughter cell, without the double antibiotic culturing, haploid cells was formed. But with the pressure, only cells containing both Chr.*ampr* and Chr.*cmr* could survive, and the diploid genomic configuration was maintained. In addition, the results of growth status of the diploid *E. coli* indicated that the diploid *E. coli* cells have a significantly slower growth rate, only half of the WT. And the transcriptome sequencing data suggested that the diploid status might affect the proliferation signaling pathways, which increased the DT and delayed the daughter‐cell separation process. These results suggested that the diploid status was not natural, which impacted the normal cell cycles, and more details were to be revealed.

Since there was only culturing method to obtain corresponding haploid and polyploid plant and yeast cells, scientific research concerning the mechanism and function of polyploidy was very limited. Meanwhile, compared with haploid strains, diploid and triploid strains maybe more robust, and many of them have been used in industrial fermentation. For example, the diploid xylose fermentation, which have an improved xylose fermentation ability and increased ethanol yield, accompanied by increased tolerance to heat, acid, ethanol, and other inhibitors.^[^ ^27^ ^]^ With the artificial diploid *E. coli*, we now have an ideal pair of objects, diploid and haploid *E. coli*, for conducting experiments to answer quite a few fundamental biological questions concerning the polyploidy. For example, it was experimentally demonstrated, for the first time, that an artificial diploid had a greater ability to survive under DNA‐damaging environments, such as the UV radiation, than the haploid form. The diploid *E. coli* is a novel artificial species with great research or application potential, which could be a major achievement in synthetic biology. Accordingly, we will make it freely available to the scientific community, and some more interesting work might be carried out with this novel research object.

## 4 Experimental Section

### Strains and Culture Conditions

*E. coli* DH5*α* was used as a cloning host. Wild‐type *E. coli* MG1655 was used in the genome duplication experiments. Strains were grown at 30 °C in Luria‐Bertani medium (LB, 1% w/v tryptone, 0.5% w/v yeast extract, and 1% w/v NaCl). Kanamycin (50 mg L^−1^), chloramphenicol (30 mg L^−1^), ampicillin (100 mg L^−1^), spectinomycin (100 mg mL^−1^), and apramycin (50 mg L^−1^) were added to the medium when appropriate. 1% w/v glucose and 2 g L^−1^ L‐arabinose were added to the culture for the repression and induction of Cas9 expression, respectively.

*E. coli* MG1655 cells with or without plasmids were grown in 50 mL of LB medium supplemented with the appropriate antibiotics at 30 °C to an OD_600_ of 0.6, and then made electrocompetent by concentrating 100‐fold and washing three times with 10% ice‐cold glycerol. Then, 50 ng of plasmid DNA or 800 ng of the editing cassette was used for electroporation. The shocked cells were resuspended in 1 mL of LB, incubated for 2 h at 30 °C, and spread on LB agar plates with the appropriate antibiotics.

### Plasmid Construction

The plasmid pgRNA was assembled using type IIS restriction enzymes‐based assembly method, Golden Gate,^[^ ^28^ ^]^ for which DNA primers were designed using J5 Device Editor.^[^ ^29^ ^]^ Inducible gRNA plasmids were constructed for guiding CRISPR/Cas9 to the target locus between *ddp*X and *dos*P on the chromosome of *E. coli* MG1655. The backbone of pgRNA was PCR amplified from pACYC184‐M,^[^ ^30^ ^]^ and the gRNA with its promoter was amplified from the plasmid pRed_Cas9_ΔpoxB300.^[^ ^31^ ^]^ The plasmid pgRNA(N20PAM) was constructed using the same method. Plasmid pRedCas9 for the inducible expression of *λ*‐Red and Cas9 was modified from pRed_Cas9_Δ*pox*B300 and assembled using the Golden Gate method.

### Construction of the Editing Cassette

Seven modularized parts were prepared with optimized 4‐nt linkers that could be processed using type IIS restriction enzymes for assembly, which included a left homologous arm (L) from the *ddp*X gene on the genome, the left half of the ampicillin resistance gene (*ampr*_L), two CRISPR/Cas9 recognition regions (N20PAM), a chloramphenicol resistance gene (*cat*), the right half of ampicillin resistance gene (*ampr*_R), and a right homologous arm (R) from the *dos*P gene on the chromosome. There were two identical sequences of 40 bp at the end of *ampr*_L and at the front of *ampr*_R, which were used to reconstruct the entire ampicillin resistance gene after CRISPR/Cas9 cleavage induced homologous recombination. A Golden Gate reaction was performed to assemble these parts into the editing cassette. The L and R homologous arms (about 500 bp each) were amplified from the genomic DNA of *E. coli* MG1655. The selection marker genes (*ampr*_L, *ampr*_R, and *cat*) with the CRISPR/Cas9 recognition region (N20PAM) were PCR‐amplified from plasmids pETDuet1 and pACYCDuet1 with the N20PAM sequence embedded in the reverse primer.

All the DNA templates were PCR‐amplified using Phusion polymerase (New England Biolabs, USA). PCR products were purified by preparative agarose gel electrophoresis using the AxyPrep DNA Gel Extraction Kit (Axygen Biosciences, USA), and the template was digested with *Dpn*I before assembly. Primers for the construction of the plasmid and editing cassette, as well as other primers are summarized in Table S2, Supporting Information.

### Chromosome Doubling Procedure

The genome editing process is illustrated in Figure 1. *E. coli* MG1655 competent cells harboring pRedCas were prepared by inducing the expression of the CRISPR‐Cas9 system and *λ*‐RED proteins using L‐arabinose. An aliquot comprising 50 µL of the competent cells was mixed with 50 ng of pgRNA and 800 ng of editing‐cassette DNA in a 2‐mm Gene Pulser cuvette (Bio‐Rad, USA). After electroporation at 2.5 kV and immediate resuspension in 1 mL of ice‐cold LB medium, the cells were incubated for 2 h at 30 °C, and then spread on LB agar plates with kanamycin, apramycin, and chloramphenicol. For each editing experiment, ten transformants were identified by colony PCR with a forward primer upstream of the left homologous arm, and a reverse primer downstream of the right homologous arm, and the expected PCR products were subjected to DNA sequencing for further confirmation. A correct clone was transferred into LB medium with kanamycin and chloramphenicol and grown overnight at 42 °C to eliminate the temperature‐sensitive pgRNA plasmid with the apramycin resistance gene. A single colony that grew on kanamycin and chloramphenicol plates but did not grow with apramycin was selected for subsequent experiments, designated as Haploid^Cm^ *E. coli* harboring the pRedCas9 plasmid. Haploid^Cm^ *E. coli* harboring pRedCas9 was then transformed with the pgRNA(N20PAM) plasmid through electroporation. Transformants were grown in LB medium at 30 °C with appropriate antibiotics for 2 h, after which 2 g L^−1^ L‐arabinose was added to induce the expression of the CRISPR‐Cas9 system and *λ*‐RED proteins. After overnight culture, a large number of dead cells appeared, and cells were transferred into fresh LB medium with ampicillin and the other three antibiotics at an inoculation ratio of 30%. After the culture recovered growth, it was spread on LB agar plates with ampicillin, kanamycin, apramycin, and chloramphenicol. To identity correctly edited clones, ten colonies were analyzed by colony PCR with three primer pairs designed to amplify the key regions as illustrated in Figure 2A, and the PCR products were subjected to DNA sequencing for further confirmation. Eventually, an artificial Diploid^Amp&Cm^ *E. coli* was created, while the reference strain Haploid^Amp^ *E. coli* was created using the same method without adding chloramphenicol. After finishing all genome modifications, all editing plasmids were eliminated by growing overnight at 42 °C. A more detailed genome editing protocol is provided in the Supporting Information.

### Separation of Single Diploid^Amp&Cm^ *Escherichia coli* Cell

In order to ensure that the tested population was uniformly grown from single‐cell, but not the mixture of more than one strain. First, the maximal dilution and plating method was adopted to obtain single‐cell formed colonies on the LB agar plate. Second, the flow cytometry method was used to separate and sort out single cells. Specifically, cells of diploid *E. coli* in the exponential phase were harvested, and the morphological characteristics were observed under a 100× oil objective using a Leica AF6000 Modular system. Next, the culture was further diluted and subjected to the flow cytometry sorting and maximal dilution plating, respectively. Using the sorting function of flow cytometry, the main distribution area of diploid *E. coli* was determined based on the cells producing forward scatter and side scatter. Before sorting, the optical path of flow cytometer was calibrated according to the instructions; the sorting pathway was calibrated according to the sorting procedure of flow cytometry, and the single cell sorting procedure was established.

### Quantification of the Relative Abundance of Reference Genes in the Diploid *Escherichia coli* Cells

To determine chromosomal copy number, a recently developed Real Time PCR approach was applied.^[^ ^14^ ^]^ First, standard PCR was used to amplify an ≈1‐kbp fragment from the *cat* gene on plasmid pACYCDuet1 and ≈1‐kbp fragments from the endogenous gene. The DNA concentration of these fragments was determined spectrophotometrically after gel purification, and the molar concentration of these DNA molecules was calculated based on their molecular weights using “‘oligo calc”’ (www.basic.northwestern.edu/biotools). Standard samples were diluted to different concentrations and used as a template for RT‐PCR. The logarithm of the molar concentration of the standard sample was plotted to the abscissa, and the measured Ct value was plotted to the ordinate to draw the standard curve. Thus, the molar concentration of an unknown sample could be obtained from the standard curve according to its Ct value. As an additional control, standard samples with known molar concentration were added as internal standards to the cell extract, and it was confirmed that the added standard was correctly quantified using this method.

### Fluorescence Imaging

For FISH, diploid *E. coli* cells were fixed in 2.5% paraformaldehyde and processed as in ref. [32]; 1‐kb probes were amplified from genomic DNA (Table S2, Supporting Information). The FISH probes were *ampr*, Alexa Fluor 488 dye and *cat*, and Alexa Fluor 594 dye (FISH Tag DNA Multicolor Kit; Invitrogen). DAPI was used to stain the genome, and the *SlowFate* Gold antifade reagent was used to improve fluorescence stability. All images were acquired under a 100× oil objective using a Leica AF6000 Modular system plus charge‐coupled device camera. Haploid^WT^, Haploid^Cm^, and Haploid^Cm^ were used as a control strains.

### De Novo Genome Sequencing and Transcriptome Sequencing of the Diploid *Escherichia coli*

Cells of the diploid *E. coli* were harvested. Total genomic DNA was then extracted with the SDS method and determined by the agarose gel electrophoresis and quantified by Qubit 2.0 Fluorometer (Thermo Scientific). Libraries for SMRT sequencing were constructed with an insert size of 10 kb using the SMRT bell TM Template kit (version 1.0). Then sequencing was performed by Novogene (Tianjin, Tianjin, China) using PacBio Sequel platform. Reads with length shorter than 500 bp were filtered afterward. To perform genome assembly, the SMRT Link v5.0.1 was used to get a preliminary assembly. Finally, arrow (2.3.3) software was used to optimize and assemble a circular genome without gap.

Total RNA of haploid and the diploid *E. coli* were extracted in the exponential phase (three independent biological replicates for each case). Then strand‐specific RNA‐seq library were prepared. Sequencing was performed on an Illumina NovaSeq 6000 platform and 150‐bp paired‐end reads were generated (Novogene, Tianjin, China). Finally, clean reads were obtained after removing reads containing adapter, reads containing N base, and reads with low quality. To estimate gene expression, reads were aligned to the genome assembled using Bowtie2 (2.3.4.3). HTSeq (v0.9.1) was then used to count the reads numbers mapped to each gene. Differential expression analysis between haploid and the diploid *E. coli* was performed using the DESeq2 R package (1.20.0). padj <0.05 and |log2(fold change)| >0 were set as the threshold for significantly differential expression.

### Investigation of the Chromosomal Stability of Diploid *Escherichia coli*

The diploid *E. coli* strain was continuously transferred in batch culture at a 1‰ inoculation ratio with or without antibiotics, and cultured overnight for 16–18 h until OD_600_ reached ≈5. A total of 11 rounds of inoculation were conducted, corresponding to ≈100 generations. The details were added to the revised manuscript. The culture of the last round was with no antibiotics spread on LB agar plates with and without antibiotics (ampicillin and chloramphenicol), while the culture with both antibiotic was spread on plates with both antibiotics. Eight colonies from the three groups were analyzed by colony PCR, using the above mentioned three sets of identification primers.

### Calculation of the Exponential Growth Rate

The growth profiles of Haploid^WT^ and Diploid^Amp&Cm^ *E. coli* were analyzed in a 48 deep multiwell plates containing LB medium with or without antibiotics (ampicillin and chloramphenicol), with a microbial growth analysis system (Jieling, China). The optical density at a wavelength of 600 nm was measured every 10 min for 25 h in total. Five independent biological replicates were performed. The growth rates during the exponential phase were evaluated according to the growth curves. The DT was calculated based on two continuous reading points in a growth curve, according to Equation (1).

(1) $$DT=\left(t_{j}-t_{i}\right)/\log_{2}\left(\frac{C_{j}}{C_{i}}\right)$$

where *C_i_* and *C_j_* represented the two OD_600_ values at two continuous time points of *t_i_* and *t_j_*, which were at intervals of either 0.5 or 1 (h) in the present study. Every four to five continuous growth rates that exhibited the largest mean and the smallest standard deviation were averaged to calculate the exponential DT for the growth curve.

### Investigation of Cell Morphology by Scanning Electron Microscopy

For SEM, cells of the wild‐type haploid *E. coli* and the diploid *E. coli* in the exponential phase were harvested, and washed three times with phosphate buffered saline (pH = 7.2). The samples were fixed for 2 h in 2.5% glutaraldehyde and post‐fixed for 1 h with 1% of osmium tetroxide. The samples were dehydrated with ethanol and dried in an automated critical point dryer (Leica EM CPD300). Then, the samples were coated with platinum and observed under a scanning microscope (Hitachi SU8010).

### Characterization of the Survival Rate of Diploid *Escherichia coli* under UV

A scheme of the UV tolerance test for characterizing the survival of diploid *E. coli* is illustrated in Figure 6. Diploid and wild‐type haploid *E. coli* cells in the exponential phase were diluted and spread on LB plates with and without antibiotics. The cells were diluted by 10^2^‐fold for exposing to UV radiation in a sterile cabinet, and the reference without UV exposure was diluted 10^5^‐fold. The SRs of the two strains were calculated using Equation (2).

(2) $$2\mathrm{SR}=N_{1}/N_{2}\times 10^{-3}\;\%$$

where *N*_1_ and *N*_2_ represented the number of clones with and without UV exposure, respectively. The experiments were done in triplicate.

## Conflict of Interest

The authors declare no conflict of interest.

## Author Contributions

P.W., D.Z., and J.L. contributed equally to this work. X.Z., C.B., and J.L. designed the research, analyzed data, and wrote the manuscript. P.W. designed the research, performed experiments, analyzed data, and wrote the manuscript. D.Z. and F.F. performed experiments and analyzed data. J.S., S.L., and Z.M. performed experiments and wrote the manuscript. C.Z., Z.D., and X.L. wrote the manuscript.

## Supporting information

Supporting Information

## Data Availability

The data that support the findings of this study are available in the supplementary material of this article.
