# Supplementary material for: Artificial Diploid Escherichia coli by a CRISPR Chromosome‐Doubling Technique
Source: Adv Sci (Weinh). 2023 Jan 15;10(7):2205855. doi: 10.1002/advs.202205855 (PMC9982549; doi:10.1002/advs.202205855)
Supplement: Supplementary file 1 — Supporting Information [file ADVS-10-2205855-s001.pdf]

ADVANCED SCIENCE
Open Access

Supporting Information

for *Adv. Sci.*, DOI 10.1002/advs.202205855

Artificial Diploid *Escherichia coli* by a CRISPR Chromosome-Doubling Technique

Pengju Wang, Dongdong Zhao, Ju Li, Junchang Su, Chunzhi Zhang, Siwei Li, Feiyu Fan, Zhubo Dai, Xiaoping Liao, Zhitao Mao, Changhao Bi and Xueli Zhang**

Supporting Information

Artificial diploid *Escherichia coli* by a CRISPR chromosome-doubling technique

Pengju Wang[1,3#], Dongdong Zhao[1,3#], Ju Li[2#], Junchang Su[1,4], Chunzhi Zhang[4], Siwei Li[1,3], Feiyu Fan[1,3], Zhubo Dai[1,3], Xiaoping Liao[1,5], Zhitao Mao[1,5], Changhao Bi[1,3] & Xueli Zhang[1,3*]*

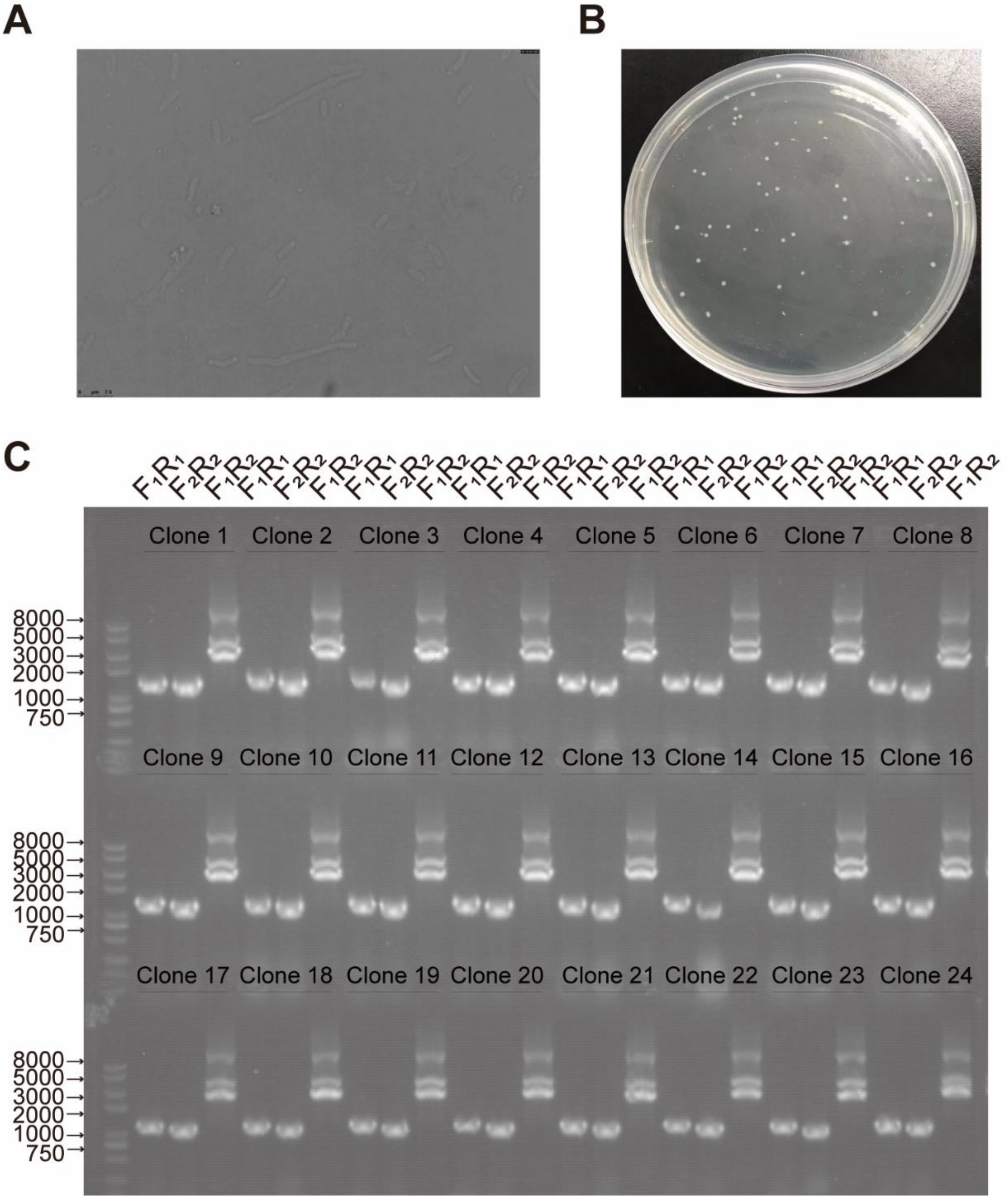

Figure S1. Separate single diploid *E. coli* cells with limiting dilution plating. (A) Microscopic observation of the diploid *E. coli* cells; (B) Diploid *E. coli* colonies derived from the single cells produced by limiting dilution plating; (C) Colony PCR analysis of the diploid *E. coli* cells produced by limiting dilution plating.

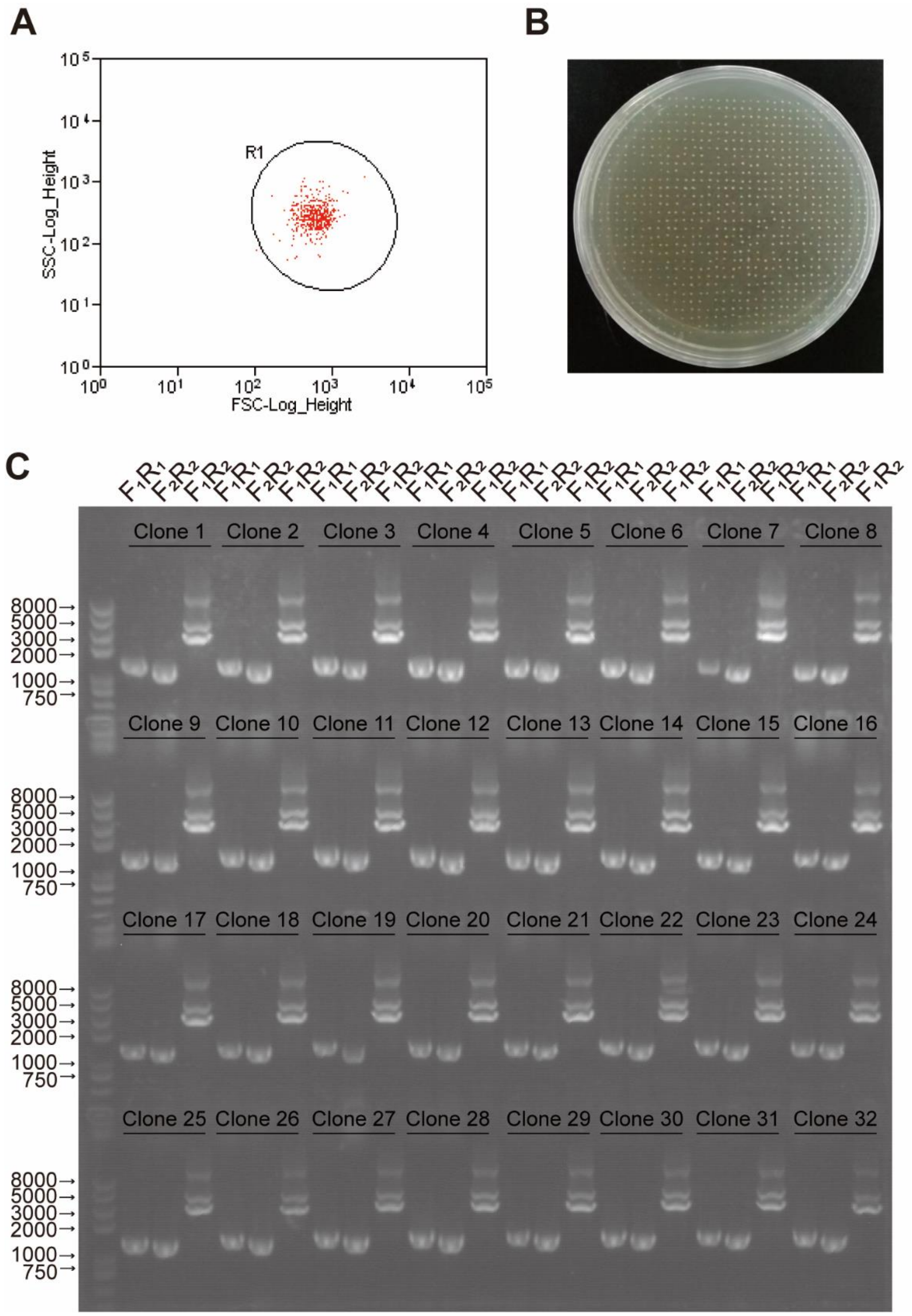

Figure S2. Separate single diploid *E. coli* cells with flow cytometry sorting. (A) Dot plots obtained via cell sorting of diploid *E. coli* cells; (B) Diploid *E. coli* colonies derived from the single cells produced by flow cytometry sorting; (C) Colony PCR analysis of the diploid *E. coli* cells produced by flow cytometry sorting.

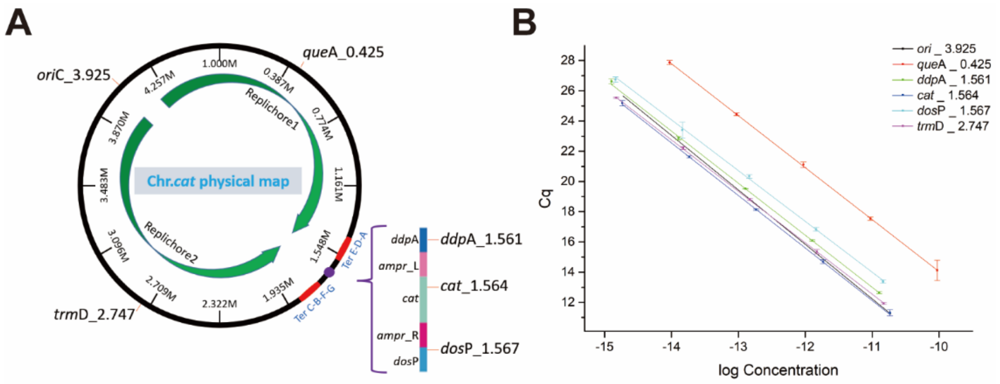

C

Gene _ locus	Equation; R^2	Haploid^{Cm}	Diploid$^{Amp\&Cm}$
*ori*C_3.925	Y = -3.60x - 27.41; R^2 = 0.9985	9.63 ± 0.66 * 10^{-13} M	9.86 ± 1.14 * 10^{-13} M
*que*A_0.425	Y = -3.44x - 23.80; R^2 = 0.9999	3.89 ± 0.23 * 10^{-13} M	4.22 ± 0.20 * 10^{-13} M
*ddp*A _1.561	Y = -3.44x - 24.89; R^2 = 0.9998	1.82 ± 0.12 * 10^{-13} M	2.22 ± 0.18 * 10^{-13} M
*cat*_1.564	Y = -3.48x - 26.11; R^2 = 0.9999	1.81 ± 0.14 * 10^{-13} M	1.01 ± 0.09 * 10^{-13} M
*dos*P_1.567	Y = -3.36x - 22.97; R^2 = 0.9995	1.94 ± 0.32 * 10^{-13} M	2.16 ± 0.24 * 10^{-13} M
*trm*D_2.747	Y = -3.40x - 24.88; R^2 = 1.0000	3.45 ± 0.65 * 10^{-13} M	3.75 ± 0.31 * 10^{-13} M

Figure S3. Confirmation of diploid *E. coli* by quantitative PCR. (A) Six genes' relative loci on the Haplid^{Cm} *E. coli* genome physical map; (B) Real-time PCR standard curves of 6 genes; (C) Table with molar concentrations of 6 genes in haploid and diploid *E. coli*.

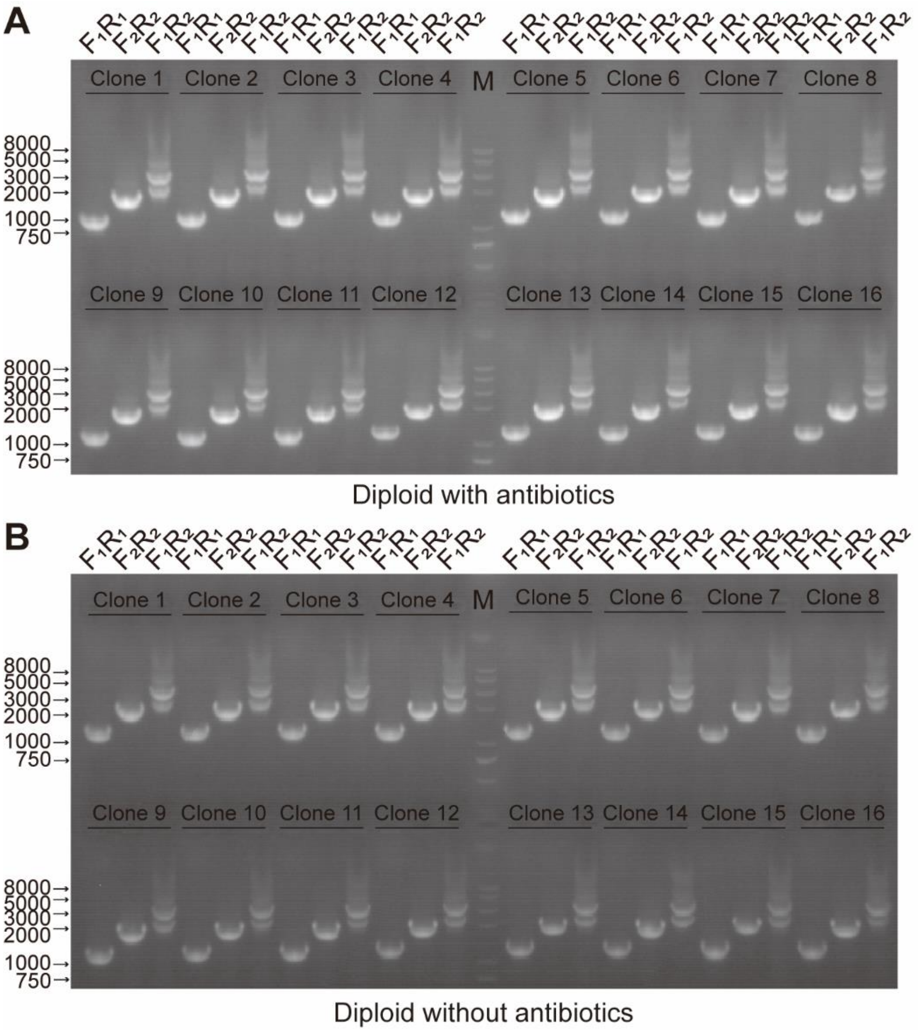

Figure S4. Colony PCR analysis of the diploid chromosomal stability. (A) Colony PCR analysis of the diploid *E. coli* cells from Deep-well Multiwell Plate with antibiotics; (B) Colony PCR analysis of the diploid *E. coli* cells from Deep-well Multiwell Plate without antibiotics.

Table S1. Plasmids and *E. coli* stains used in this study

Name	Description
**Plasmids**	
pgRNA	expression of a gRNA guiding CRISPR/Cas9 to a target locus (between *ddp*X and *dos*P) on Haploid^{WT} *E. coli* genome
pgRNA(N20PAM)	expression of a gRNA guiding CRISPR/Cas9 to two N20PAM regions of the editing cassette on Haploid^{Cm} *E. coli* genome
pRedCas9	expression of CRISPR-Cas9 system and λ-RED proteins
**Strains**	
Haploid^{WT} *E. coli*	*E. coli* MG1655 (Wild type stain)
Haploid^{Cm} *E. coli*	*E. coli* MG1655 derivative with Chr.Cm
Haploid^{Amp} *E. coli*	*E. coli* MG1655 derivative with Chr.Amp
Haploid$^{Amp\&Cm}$ *E. coli*	*E. coli* MG1655 derivative with Chr.Amp&Cm

Table S2. Primers used in this study

Name	Sequence	Description
pgRNAF	ccaggtctcatagcaagttaaggaaggaatctccgttttagagctag	construction of pgRNA plasmid
pgRNAR	ccaggtctcagctaagatctgactcc	
pgRNA(N20PAM)F	ccaggtctcatagctagtccatcgaaccgaagtagttttagagctag	construction of pgRNA(N20PAM) plasmid
pgRNA(N20PAM)R	ccaggtctcagctaagatctgactcc	
LF	accgtcacatcaacaacatattgc	construction of editing cassette
LR	ccaggtctcagtcgtgacactgtcacttaaatactgtg	
*ampr*_LF	ccaggtctcacgacgtcaggtggcacttttcggggaaatg	
*ampr*_LR	ccaggtctcagccatacttcggttcgatggactagtgc	
*cat*F	ccaggtctcatggccagcatgtgcagctccatcagcaaaag	
*cat*R	ccaggtctcataccttacgccccgccctgccactcatc	
*ampr*_RF	ccaggtctcaggtagtccatcgaaccgaagtatggaccg	
*ampr*_RR	ccaggtctcattcgaagatcctttgatcttttctacggggtctg	
RF	ccaggtctcacgaagttaaggaaggaatctcccggaatcgtagc	
RR	gcacggaaagcatgatgatggaacacgataccg	
F_1	tatcgaggatgttcccgtgctcatcacgaag	confirm of Diploid$^{Amp\&Cm}$ *E. coli*
R_1	tccagtgtcaacaccgccagagataacagtc	
F_2	atcagtaagttggcagcatcacccgacgc	
R_2	gtcggcgctgcactttcgcagtaatcaac	
*ori*CF	tggcacggaacttcagtcccatttg	amplification of the standard fragment
*ori*CR	ggtgccgaatatgtagcagaacacc	
*que*AF	atgcgcgttaccgatttctcc	
*que*AR	ttactccccgacgcgctc	
*ddp*AF	tttcatagaacgagcggttacccggcagac	
*ddp*AR	cgttgaaggcgatctggcaagtagctggaaag	
*cat*F	ccagcatgtgcagctccatcagcaaaag	
*cat*R	ccttacgccccgccctgccactcatc	
*dos*PF	ccattgcattgtgagcagtggagag	
*dos*PR	tgtgcagtgcaatcgcgcatttacc	
*trm*DF	acggtatcacccggacggaaggaag	
*trm*DR	atgcggcgaacctgctgacgaattg	
*ori*CqF	accggttgatccaagcttcc	Used for RT-PCR analyses
*ori*CqR	agatccggcagaagaatggc	
*que*AqF	acaatgaccgcgcgccac	
*que*AqR	cgatatacggcggcagcg	
*ddp*AqF	tgtgtcgccagagcaatagg	
*ddp*AqR	gaaagccaaagccgaatggg	
*cat*qF	caacagtgccgttgatcgtg	
*cat*qR	gggtgatgctgccaacttac	
*dos*PqF	gtgcgaacagcgaaacatgc	
*dos*PqR	atattctcgccgccatgtgc	
*trm*DqF	cccagcgactgtttcaaacg	
*trm*DqR	tggattgcccgcactatacg	

Table S3. The schematic and sequences of the editting cassette used in this study.

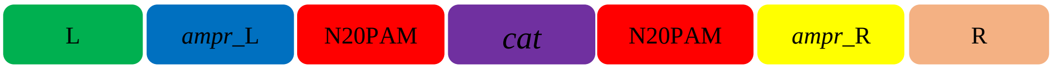

accgtcacatcaacaacatattgcgggtctgggcaggcttgccacaacatcgcctgtgcttgttgtgggcgatacgcatcgtaaatcaccagttgtaa
ccctgacagctgggcgatgctgatacttttcgccagcgcggtaatcgcatccttgtgtaacagacaacgcgcttgctgataaatagctttacctgtgat
gttatcagcgcaggcgtatttcaattcgatctccagatcagggaagattacggctaaatcaaccagttcggtggtatccgacataaacggttcctgtata
agacaaaaattgctgcgctttccgcttatgcagatctcatgccatgccgggataagcgccagaatgctggcttaaagttatatttttaacttttgatcaac
atttgtgcagcgtagtgcagttttggtgcaagaggggaagttaaggaaggaatctcccggaatcgtagctgaaatcacagtatttaagtgacagtgtc
acgacgtcaggtggcacttttcggggaaatgtgcgcggaacccccatttgtttatttttctaaatacattcaaatatgtatccgctcatgagacaataacc
ctgataaatgcttcaataatattgaaaaaggaagagtatgagtattcaacatttccgtgtcgcccttattcccttttttgcggcattttgccttcctgtttttgct
cacccagaaacgctggtgaaagtaaaagatgctgaagatcagttgggtgcacgagtgggttacatcgaactggatctcaacagcggtaagatcctt
gagagttttcgccccgaagaacgttttccaatgatgagcacttttaaagttctgctatgtggcgcggtattatcccgtattgacgccgggcaagagcaa
ctcggtcgccgcatacactattctcagaatgacttggttgagtactcaccagtcacagaaaagcatcttacggatggcatgacagtaagagaattatgc
agtgctgccataaccatgagtgataacactgcggccaacttacttctgacaacgatcggaggaccgaaggagctaaccgcttttttgcacaacatgg
gggatcatgcactagtccatcgaaccgaagtatggccagcatgtgcagctccatcagcaaaaggggatgataagtttatcaccaccgactatttgca
acagtgccgttgatcgtgctatgatcgactgttatctctggcggtgttgacactggagcacctcaaaaacaccatcatacactaaatcagtaagttggc
agcatcacccgacgcactttgcgccgaataaatacctgtgacggaagatcacttcgcagaataaataaatcctggtgtccctgttgataccgggaag
ccctgggccaacttttggcgaaaatgagacgttgatcggcacgtaagaggttccaactttcaccataatgaaataagatcactaccgggcgtattttttg
agttatcgagattttcaggagctaaggaagctaaaatggagaaaaaaatcactggatataccaccgttgatatatcccaatggcatcgtaaagaacatt
ttgaggcatttcagtcagttgctcaatgtacctataaccagaccgttcagctggatattacggcctttttaaagaccgtaaagaaaaataagcacaagttt
tatccggcctttattcacattcttgcccgcctgatgaatgctcatccggaattccgtatggcaatgaaagacggtgagctggtgatatgggatagtgttc
acccttgttacaccgttttccatgagcaaactgaaacgttttcatcgctctggagtgaataccacgacgatttccggcagtttctacacatatattcgcaa
gatgtggcgtgttacggtgaaaacctggcctatttccctaaagggtttattgagaatatgtttttcgtctcagccaatccctgggtgagtttcaccagttttg
atttaaacgtggccaatatggacaacttcttcgcccccgttttcaccatgggcaaatattatacgcaaggcgacaaggtgctgatgccgctggcgattc
aggttcatcatgccgtctgtgatggcttccatgtcggcagaatgcttaatgaattacaacagtactgcgatgagtggcagggcggggcgtaaggtagt
ccatcgaaccgaagtatggaccggaaggagctaaccgcttttttgcacaacatgggggatcatgtaactcgccttgatcgttgggaaccggagctga
atgaagccataccaaacgacgagcgtgacaccacgatgcctgtagcaatggcaacaacgttgcgcaaactattaactggcgaactacttactctagc
ttcccggcaacaattaatagactggatggaggcggataaagttgcaggaccacttctgcgctcggcccttccggctggctggtttattgctgataaatc
tggagccggtgagcgtgggtctcgcggtatcattgcagcactggggccagatggtaagccctcccgtatcgtagttatctacacgacggggagtca
ggcaactatggatgaacgaaatagacagatcgctgagataggtgcctcactgattaagcattggtaactgtcagaccaagtttactcatatatactttag
attgatttaaaacttcatttttaatttaaaaggatctaggtgaagatcctttttgataatctcatgaccaaaatcccttaacgtgagttttcgttccactgagcg
tcagaccccgtagaaaagatcaaaggatcttcgaaaaggaaggaatctcccggaatcgtagctgaaatcacagtatttaagtgacagtgtcacgtta
aatgaaaacccgcgagtgcgggcgagaggaatttgtcagattttcagcggtaacacgctgctcatccagcctggaatttcttcggcgggtaggggg
cgggaaaagaaatatccctgaataacgcgacagtggatcttgcgtagcatctcaaattgctctttggtttcgacgccttccgccacgacggttaaattg
aggctttgcccaatgctggtaatggcttcaagtaaggcaaggatgcgtttttcggtcagacaacgatcgacaaaacttttgtcaattttgatttccgttac
cggaagactgactaagcgggataatccggaaaagcccgtaccaaaatcatctaccgataagcccacgcccatatcacgcaggatctgaatgcgctt
aaagatttcggtatcgtgttccatcatcatgctttccgtgatttctaccgtcagctggtggccgtcaataccccaggcgtgcattgcatcagacacctgat
taggcagttgattactgcgaaagtgcagcgccgacaagttcacggataacgccgggatatgaatattctggctacgccattctgctaactgacggca
agcttccgcgatgacccagcgcccaatattttcgatttcaccaatctcttctgcgagagggtg

Table S4. The sequences of clone PCR products used to identity the diploid *E. coli* strain.

F_1R_1 >1320 bp

tatcgaggatgttcccgtgctcatcacgaagcgtcaggtcgatcgccgtgccacggctgtgattagaaccgaccgtcacatcaacaaca
tattgcgggtctgggcaggcttgccacaacatcgcctgtgcttgttgtgggcgatacgcatcgtaaatcaccagttgtaaccctgacagc
tgggcgatgctgatacttttcgccagcgcggtaatcgcatccttgtgtaacagacaacgcgcttgctgataaatagctttacctgtgatgtt
atcagcgcaggcgtatttcaattcgatctccagatcagggaagattacggctaaatcaaccagttcggtggtatccgacataaacggttc
ctgtataagacaaaaattgctgcgctttccgcttatgcagatctcatgccatgccgggataagcgccagaatgctggcttaaagttatatttt
ttaacttttgatcaacatttgtgcagcgtagtgcagttttggtgcaagaggggaagttaaggaaggaatctcccggaatcgtagctgaaat
cacagtatttaagtgacagtgtcacgacgtcaggtggcacttttcggggaaatgtgcgcggaacccccatttgtttatttttctaaatacatt
caaatatgtatccgctcatgagacaataaccctgataaatgcttcaataatattgaaaaaggaagagtatgagtattcaacatttccgtgtc
gcccttattcccttttttgcggcattttgccttcctgtttttgctcacccagaaacgctggtgaaagtaaaagatgctgaagatcagttgggtg
cacgagtgggttacatcgaactggatctcaacagcggtaagatccttgagagttttcgccccgaagaacgttttccaatgatgagcacttt
taaagttctgctatgtggcgcggtattatcccgtattgacgccgggcaagagcaactcggtcgccgcatacactattctcagaatgacttg
gttgagtactcaccagtcacagaaaagcatcttacggatggcatgacagtaagagaattatgcagtgctgccataaccatgagtgataa
cactgcggccaacttacttctgacaacgatcggaggaccgaaggagctaaccgcttttttgcacaacatgggggatcatgcactagtcc
atcgaaccgaagtatggccagcatgtgcagctccatcagcaaaaggggatgataagtttatcaccaccgactatttgcaacagtgccgt
tgatcgtgctatgatcgactgttatctctggcggtgttgacactgga

F_2R_2 >2134 bp

atcagtaagttggcagcatcacccgacgcactttgcgccgaataaatacctgtgacggaagatcacttcgcagaataaataaatcctggt
gtccctgttgataccgggaagccctgggccaacttttggcgaaaatgagacgttgatcggcacgtaagaggttccaactttcaccataat
gaaataagatcactaccgggcgtattttttgagttatcgagattttcaggagctaaggaagctaaaatggagaaaaaaatcactggatata
ccaccgttgatatatcccaatggcatcgtaaagaacattttgaggcatttcagtcagttgctcaatgtacctataaccagaccgttcagctg
gatattacggcctttttaaagaccgtaaagaaaaataagcacaagttttatccggcctttattcacattcttgcccgcctgatgaatgctcatc
cggaattccgtatggcaatgaaagacggtgagctggtgatatgggatagtgttcacccttgttacaccgttttccatgagcaaactgaaac
gttttcatcgctctggagtgaataccacgacgatttccggcagtttctacacatatattcgcaagatgtggcgtgttacggtgaaaacctgg
cctatttccctaaagggtttattgagaatatgtttttcgtctcagccaatccctgggtgagtttcaccagttttgatttaaacgtggccaatatg
gacaacttcttcgcccccgttttcaccatgggcaaatattatacgcaaggcgacaaggtgctgatgccgctggcgattcaggttcatcat
gccgtctgtgatggcttccatgtcggcagaatgcttaatgaattacaacagtactgcgatgagtggcagggcggggcgtaaggtagtcc
atcgaaccgaagtatggaccggaaggagctaaccgcttttttgcacaacatgggggatcatgtaactcgccttgatcgttgggaaccgg
agctgaatgaagccataccaaacgacgagcgtgacaccacgatgcctgtagcaatggcaacaacgttgcgcaaactattaactggcg
aactacttactctagcttcccggcaacaattaatagactggatggaggcggataaagttgcaggaccacttctgcgctcggcccttccgg
ctggctggtttattgctgataaatctggagccggtgagcgtgggtctcgcggtatcattgcagcactggggccagatggtaagccctcc
cgtatcgtagttatctacacgacggggagtcaggcaactatggatgaacgaaatagacagatcgctgagataggtgcctcactgattaa
gcattggtaactgtcagaccaagtttactcatatatactttagattgatttaaaacttcatttttaatttaaaaggatctaggtgaagatcctttttt
gataatctcatgaccaaaatcccttaacgtgagttttcgttccactgagcgtcagaccccgtagaaaagatcaaaggatcttcgaagttaa
ggaaggaatctcccggaatcgtagctgaaatcacagtatttaagtgacagtgtcacgttaaatgaaaacccgcgagtgcgggcgagag
gaatttgtcagattttcagcggtaacacgctgctcatccagcctggaatttcttcggcgggtagggggcgggaaaagaaatatccctgaa
taacgcgacagtggatcttgcgtagcatctcaaattgctctttggtttcgacgccttccgccacgacggttaaattgaggctttgcccaatg
ctggtaatggcttcaagtaaggcaaggatgcgtttttcggtcagacaacgatcgacaaaacttttgtcaattttgatttccgttaccggaag
actgactaagcgggataatccggaaaagcccgtaccaaaatcatctaccgataagcccacgcccatatcacgcaggatctgaatgcgc
ttaaagatttcggtatcgtgttccatcatcatgctttccgtgatttctaccgtcagctggtggccgtcaataccccaggcgtgcattgcatca
gacacctgattaggcagttgattactgcgaaagtgcagcgccgac

$F_1R_2^{WT}$ >1102 bp

Tatcgaggatgttcccgtgctcatcacgaagcgtcaggtcgatcgccgtgccacggctgtgattagaaccgaccgtcacatcaacaac
atattgcgggtctgggcaggcttgccacaacatcgcctgtgcttgttgtgggcgatacgcatcgtaaatcaccagttgtaaccctgacag
ctgggcgatgctgatacttttcgccagcgcggtaatcgcatccttgtgtaacagacaacgcgcttgctgataaatagctttacctgtgatgt
tatcagcgcaggcgtatttcaattcgatctccagatcagggaagattacggctaaatcaaccagttcggtggtatccgacataaacggttc
ctgtataagacaaaaattgctgcgctttccgcttatgcagatctcatgccatgccgggataagcgccagaatgctggcttaaagttatatttt

ttaacttttgatcaacatttgtgcagcgtagtgcagttttggtgcaagaggggaagttaaggaaggaatctcccggaatcgtagctgaaat
cacagtatttaagtgacagtgtcacgttaaatgaaaacccgcgagtgcgggcgagaggaatttgtcagattttcagcggtaacacgctg
ctcatccagcctggaatttcttcggcgggtagggggcgggaaaagaaatatccctgaataacgcgacagtggatcttgcgtagcatctc
aaattgctctttggtttcgacgccttccgccacgacggttaaattgaggctttgcccaatgctggtaatggcttcaagtaaggcaaggatg
cgtttttcggtcagacaacgatcgacaaaacttttgtcaattttgatttccgttaccggaagactgactaagcgggataatccggaaaagc
ccgtaccaaaatcatctaccgataagcccacgcccatatcacgcaggatctgaatgcgcttaaagatttcggtatcgtgttccatcatcat
gctttccgtgatttctaccgtcagctggtggccgtcaataccccaggcgtgcattgcatcagacacctgattaggcagttgattactgcga
aagtgcagcgccgac

$F_1R_2^{Amp}$ >2337 bp
tatcgaggatgttcccgtgctcatcacgaagcgtcaggtcgatcgccgtgccacggctgtgattagaaccgaccgtcacatcaacaaca
tattgcgggtctgggcaggcttgccacaacatcgcctgtgcttgttgtgggcgatacgcatcgtaaatcaccagttgtaaccctgacagc
tgggcgatgctgatacttttcgccagcgcggtaatcgcatccttgtgtaacagacaacgcgcttgctgataaatagctttacctgtgatgtt
atcagcgcaggcgtatttcaattcgatctccagatcagggaagattacggctaaatcaaccagttcggtggtatccgacataaacggttc
ctgtataagacaaaaattgctgcgctttccgcttatgcagatctcatgccatgccgggataagcgccagaatgctggcttaaagttatattt
ttaacttttgatcaacatttgtgcagcgtagtgcagttttggtgcaagaggggaagttaaggaaggaatctcccggaatcgtagctgaaat
cacagtatttaagtgacagtgtcacgacgtcaggtggcacttttcggggaaatgtgcgcggaacccctatttgtttatttttctaaatacatt
caaatatgtatccgctcatgagacaataaccctgataaatgcttcaataatattgaaaaaggaagagtatgagtattcaacatttccgtgtc
gcccttattcccttttttgcggcattttgccttcctgtttttgctcacccagaaacgctggtgaaagtaaaagatgctgaagatcagttgggtg
cacgagtgggttacatcgaactggatctcaacagcggtaagatccttgagagttttcgccccgaagaacgttttccaatgatgagcacttt
taaagttctgctatgtggcgcggtattatcccgtattgacgccgggcaagagcaactcggtcgccgcatacactattctcagaatgacttg
gttgagtactcaccagtcacagaaaagcatcttacggatggcatgacagtaagagaattatgcagtgctgccataaccatgagtgataa
cactgcggccaacttacttctgacaacgatcggaggaccgaaggagctaaccgcttttttgcacaacatgggggatcatgcacgtaact
cgccttgatcgttgggaaccggagctgaatgaagccataccaaacgacgagcgtgacaccacgatgcctgtagcaatggcaacaac
gttgcgcaaactattaactggcgaactacttactctagcttcccggcaacaattaatagactggatggaggcggataaagttgcaggacc
acttctgcgctcggcccttccggctggctggtttattgctgataaatctggagccggtgagcgtgggtctcgcggtatcattgcagcactg
gggccagatggtaagccctcccgtatcgtagttatctacacgacggggagtcaggcaactatggatgaacgaaatagacagatcgctg
agataggtgcctcactgattaagcattggtaactgtcagaccaagtttactcatatatactttagattgatttaaaacttcatttttaatttaaaa
ggatctaggtgaagatcctttttgataatctcatgaccaaaatcccttaacgtgagttttcgttccactgagcgtcagaccccgtagaaaag
atcaaaggatcttcgaagttaaggaaggaatctcccggaatcgtagctgaaatcacagtatttaagtgacagtgtcacgttaaatgaaaa
cccgcgagtgcgggcgagaggaatttgtcagattttcagcggtaacacgctgctcatccagcctggaatttcttcggcgggtaggggg
cgggaaaagaaatatccctgaataacgcgacagtggatcttgcgtagcatctcaaattgctctttggtttcgacgccttccgccacgacg
gttaaattgaggctttgcccaatgctggtaatggcttcaagtaaggcaaggatgcgtttttcggtcagacaacgatcgacaaaacttttgtc
aattttgatttccgttaccggaagactgactaagcgggataatccggaaaagcccgtaccaaaatcatctaccgataagcccacgcccat
atcacgcaggatctgaatgcgcttaaagatttcggtatcgtgttccatcatcatgctttccgtgatttctaccgtcagctggtggccgtcaat
accccaggcgtgcattgcatcagacacctgattaggcagttgattactgcgaaagtgcagcgccgac

$F_1R_2^{Cm}$ >3482 bp
tatcgaggatgttcccgtgctcatcacgaagcgtcaggtcgatcgccgtgccacggctgtgattagaaccgaccgtcacatcaacaaca
tattgcgggtctgggcaggcttgccacaacatcgcctgtgcttgttgtgggcgatacgcatcgtaaatcaccagttgtaaccctgacagc
tgggcgatgctgatacttttcgccagcgcggtaatcgcatccttgtgtaacagacaacgcgcttgctgataaatagctttacctgtgatgtt
atcagcgcaggcgtatttcaattcgatctccagatcagggaagattacggctaaatcaaccagttcggtggtatccgacataaacggttc
ctgtataagacaaaaattgctgcgctttccgcttatgcagatctcatgccatgccgggataagcgccagaatgctggcttaaagttatattt
ttaacttttgatcaacatttgtgcagcgtagtgcagttttggtgcaagaggggaagttaaggaaggaatctcccggaatcgtagctgaaat
cacagtatttaagtgacagtgtcacgacgtcaggtggcacttttcggggaaatgtgcgcggaacccctatttgtttatttttctaaatacatt
caaatatgtatccgctcatgagacaataaccctgataaatgcttcaataatattgaaaaaggaagagtatgagtattcaacatttccgtgtc
gcccttattcccttttttgcggcattttgccttcctgtttttgctcacccagaaacgctggtgaaagtaaaagatgctgaagatcagttgggtg
cacgagtgggttacatcgaactggatctcaacagcggtaagatccttgagagttttcgccccgaagaacgttttccaatgatgagcacttt
taaagttctgctatgtggcgcggtattatcccgtattgacgccgggcaagagcaactcggtcgccgcatacactattctcagaatgacttg
gttgagtactcaccagtcacagaaaagcatcttacggatggcatgacagtaagagaattatgcagtgctgccataaccatgagtgataa
cactgcggccaacttacttctgacaacgatcggaggaccgaaggagctaaccgcttttttgcacaacatgggggatcatgcactagtcc

atcgaaccgaagtatggccagcatgtgcagctccatcagcaaaaggggatgataagtttatcaccaccgactatttgcaacagtgccgt
tgatcgtgctatgatcgactgttatctctggcggtgttgacactggagcacctcaaaaacaccatcatacactaaatcagtaagttggcag
catcacccgacgcactttgcgccgaataaatacctgtgacggaagatcacttcgcagaataaataaatcctggtgtccctgttgataccg
ggaagccctgggccaacttttggcgaaaatgagacgttgatcggcacgtaagaggttccaactttcaccataatgaaataagatcacta
ccgggcgtatttttttgagttatcgagattttcaggagctaaggaagctaaaatggagaaaaaaatcactggatataccaccgttgatatatc
ccaatggcatcgtaaagaacattttgaggcatttcagtcagttgctcaatgtacctataaccagaccgttcagctggatattacggccttttt
aaagaccgtaaagaaaaataagcacaagttttatccggcctttattcacattcttgcccgcctgatgaatgctcatccggaattccgtatgg
caatgaaagacggtgagctggtgatatgggatagtgttcacccttgttacaccgttttccatgagcaaactgaaacgttttcatcgctctgg
agtgaataccacgacgatttccggcagtttctacacatatattcgcaagatgtggcgtgttacggtgaaaacctggcctatttccctaaag
ggtttattgagaatatgtttttcgtctcagccaatccctgggtgagtttcaccagttttgatttaaacgtggccaatatggacaacttcttcgcc
cccgttttcaccatgggcaaatattatacgcaaggcgacaaggtgctgatgccgctggcgattcaggttcatcatgccgtctgtgatggc
ttccatgtcggcagaatgcttaatgaattacaacagtactgcgatgagtggcagggcggggcgtaaggtagtccatcgaaccgaagtat
ggaccggaaggagctaaccgcttttttgcacaacatgggggatcatgtaactcgccttgatcgttgggaaccggagctgaatgaagcc
ataccaaacgacgagcgtgacaccacgatgcctgtagcaatggcaacaacgttgcgcaaactattaactggcgaactacttactctagc
ttcccggcaacaattaatagactggatggaggcggataaagttgcaggaccacttctgcgctcggcccttccggctggctggtttattgc
tgataaatctggagccggtgagcgtgggtctcgcggtatcattgcagcactggggccagatggtaagccctcccgtatcgtagttatcta
cacgacggggagtcaggcaactatggatgaacgaaatagacagatcgctgagataggtgcctcactgattaagcattggtaactgtca
gaccaagtttactcatatatactttagattgatttaaaacttcatttttaatttaaaaggatctaggtgaagatcctttttgataatctcatgacca
aaatcccttaacgtgagttttcgttccactgagcgtcagaccccgtagaaaagatcaaaggatcttcgaagttaaggaaggaatctcccg
gaatcgtagctgaaatcacagtatttaagtgacagtgtcacgttaaatgaaaacccgcgagtgcgggcgagaggaatttgtcagattttc
agcggtaacacgctgctcatccagcctggaatttcttcggcgggtagggggcgggaaaagaaatatccctgaataacgcgacagtgg
atcttgcgtagcatctcaaattgctctttggtttcgacgccttccgccacgacggttaaattgaggctttgcccaatgctggtaatggcttca
agtaaggcaaggatgcgtttttcggtcagacaacgatcgacaaaacttttgtcaattttgatttccgttaccggaagactgactaagcggg
ataatccggaaaagcccgtaccaaaatcatctaccgataagcccacgcccatatcacgcaggatctgaatgcgcttaaagatttcggtat
cgtgttccatcatcatgctttccgtgatttctaccgtcagctggtggccgtcaataccccaggcgtgcattgcatcagacacctgattaggc
agttgattactgcgaaagtgcagcgccgac

Table S5. The sequences of standard fragments for RT-PCR.

*ori*CF/R >1228 bp
tggcacggaacttcagtcccatttgggtaacagcaccgaccacgcgatcgttttcgacaataagatcttcaaccgcctgctggaagatca
tcaggttcggttggttctccagcgccgtacgtaccgcctgacggtagagcacacgatccgcctgagctcgggtagcgcgaaccgccg
gtcctttgcttgcgtttagtatcctaaactggatacccgcctgatcgatcgctttcgccatcagaccgccgagtgcatccacttcttttacca
gatgtcccttcccaataccgccgatcgccgggttgcagctcatctgccccagagtgtcgatattgtgtgtcaaaagcagagtctgttgac
ccatacgcgccgcggccatcgcggcctcggtgcctgcatgacccccgccaatgatgatgacgtcaaaaggatccggataaaacatg
gtgattgcctcgcataacgcggtatgaaaatggattgaagcccgggccgtggattctactcaactttgtcggcttgagaaagacctggg
atcctgggtattaaaaagaagatctatttatttagagatctgttctattgtgatctcttattaggatcgcactgccctgtggataacaaggatcc
ggcttttaagatcaacaacctggaaaggatcattaactgtgaatgatcggtgatcctggaccgtataagctgggatcagaatgaggggtt
atacacaactcaaaaactgaacaacagttgttctttggataactaccggttgatccaagcttcctgacagagttatccacagtagatcgca
cgatctgtatacttatttgagtaaattaacccacgatcccagccattcttctgccggatcttccggaatgtcgtgatcaagaatgttgatcttc
agtgtttcgcctgtctgttttgcaccggaatttttgagttctgcctcgagtttatcgatagccccacaaaaggtgtcatattcacgactgccaa
taccgattgcgccaaagcggactgcagaaagatcgggcttctgttcctgcaatgcttcatagaaaggagaaaggttgtccggaatatct
ccggcaccgtgggtggagctgataaccagccagatccctgaggcaggtaaatcttctaacagcggaccgtgcagcgtttcggtggta
aaacccgcctcttccagcttttcagccaggtgttctgctacatattcggcacc

*que*AF/R >1071 bp
atgcgcgttaccgatttctcctttgaattgcccgaatccctgattgcccactatcccatgcctgaacgcagtagctgtcgtttactgtcgctg
gacgggccgacgggcgcgctgacgcacggtactttcaccgatttacttgataagctcaaccccggcgatcttctggtttttaataatacc
cgcgtgatcccggcgcgcctgtttgggcgtaaagccagcggcggcaagattgaagtgctggttgaacggatgctcgacgacaaacg
cattcttgcgcatattcgcgcctcgaaagcgccaaaacctggcgcagaactgctgctgggcgatgacgaaagtattaacgcaacaatg
accgcgcgccacggcgcactgtttgaagtcgaatttaatgatgaacgctcggtgctggatattctcaacagcatcggccatatgccgct
gccgccgtatatcgaccgtccggacgaagacgctgaccgcgaactttatcaaaccgtttatagcgaaaaaccgggcgcggttgcagc
cccgaccgcaggtctgcattttgacgagcctttgctggaaaaaattgcgcgccaaaggcgtggagatggcgtttgtgacgttgcacgttg
gtgcgggcaccttccagccggtgcgcgtcgacaccattgaagatcacatcatgcactcggaatacgctgaagtaccgcaggatgtggt
agacgcggtactggcggcgaaagcgcgcggtaaccgggtgattgcggttggcaccacttcagtacgttcgctggaaagcgcggctc
aggcagcgaaaaacgatctcattgaaccgttcttcgacgatacccaaatctttatctatccgggcttccagtacaaagtggtcgatgcgct
ggtgacgaacttccacttgccagagtcgacgctgattatgctggtttcggcctttgccggttatcaacacaccatgaacgcctataaagca
gcggtagaagagaaatatcgctttttagttacggtgatgcgatgtttatcacgtacaatccgcaggcaattaatgagcgcgtcggggagt
aa

*ddp*AF/R >1052 bp
cgcattgcgcagtaacttatcgacctcactgttttcatagaacgagcggttacccggcagaccttttttgtctgactcaaaccagtaattcat
aaacatatacgggtcggcaaaatccggactccagttgccaatcgcaatgtcgtaatcacctttacccactctgtcgcgcatggtggcgtt
cgccagcttttccagcttcacaatgatgcccagcttgttgagactggattgtgtcgccagagcaataggctcccagttcggatcgttatcg
gagtagagaaacgtcaggctggtgggtttgctcgtcactttatcccattcggctttggctttcgtttcgtcatggttgtattgcattgccgtcg
catcgtagccccacatgccttccggaatcgggccgcgcatctgttttccgttaccactcagaatgccgttaaccatgccctgataatcggt
agaccaggaaatggcccgacgcagatccgcctgattaagaggcgctttgctgttattcagatacagataggtaacgcgcagtgacgga
tactctgccacattgactttattttcctgcttcagggcgttgagttgatccaccggcagcgcatcggcaatgtcaatgtcgccacgggaga
gctgcaggcgacgggaggcactttcaccaataatttttaccgatacccgtttgaagttcggtttattgcctgggtaatgcggatttggcacc
agaactaattgctgaccttttttgccagcttttcagcataaatggtccggaaccggcggtattttgcgcgaggaagccgcgagcatcatcc
gctgcatgttcctttaagaccgccggattgataatggatgcaccgtcattcgccagcgtgtagaggaacggtgcgaatggttggctaag
ggtaaacttcaccgtatgttcgtcgggagcatcaatctttaaatctttgggaaatgcttctgctggccccctggccgatttttagtagccgctc
aaaagaaagttttactgcttcggcagtgacaggtgtgccatcggcaaatt

*cat*F/R >1055 bp
ccagcatgtgcagctccatcagcaaaaggggatgataagtttatcaccaccgactatttgcaacagtgccgttgatcgtgctatgatcga
ctgttatctctggcggtgttgacactggagcacctcaaaaacaccatcatacactaaatcagtaagttggcagcatcacccgacgcacttt
gcgccgaataaatacctgtgacggaagatcacttcgcagaataaataaatcctggtgtccctgttgataccgggaagccctgggccaac

ttttggcgaaaatgagacgttgatcggcacgtaagaggttccaactttcaccataatgaaataagatcactaccgggcgtatttttgagtt
atcgagattttcaggagctaaggaagctaaaatggagaaaaaaatcactggatataccaccgttgatatatcccaatggcatcgtaaaga
acattttgaggcatttcagtcagttgctcaatgtacctataaccagaccgttcagctggatattacggcctttttaaagaccgtaaagaaaaa
taagcacaagttttatccggcctttattcacattcttgcccgcctgatgaatgctcatccggaattccgtatggcaatgaaagacggtgagc
tggtgatatgggatagtgttcacccttgttacaccgttttccatgagcaaactgaaacgttttcatcgctctggagtgaataccacgacgatt
tccggcagtttctacacatatattcgcaagatgtggcgtgttacggtgaaaacctggcctatttccctaaagggtttattgagaatatgttttt
cgtctcagccaatccctgggtgagtttcaccagttttgatttaaacgtggccaatatggacaacttcttcgcccccgttttcaccatgggca
aatattatacgcaaggcgacaaggtgctgatgccgctggcgattcaggttcatcatgccgtctgtgatggcttccatgtcggcagaatgc
ttaatgaattacaacagtactgcgatgagtggcagggcggggcgtaagg

*dos*PF/R >1076 bp
ccattgcattgtgagcagtggagagcaagtaatcgcggtttttacccaggtcgtagctgatgccaatactcaaggtaagcgggaagggt
ttatcgtcaatcattatcggcttgctgaccacattccgtagctcatcggcgatttgggtaatgttactgacgtcgttttcgaggctcacgagg
acaaactgcgtaccttcgatacgacagagatactgatccggtttgagtttttcacgaaagcgattgaccacttccagcaatgcctgatcgg
cccacgcatagccaaggctatcaatcacatcctgaatatggtcaacaccgatgagatacaccacgggagagacggctttgtcgaccag
gtcatcgaggtaattgtgcaggttattgcgatttggcagaccggtcatcggatcaaattggatgagttgttcaatatgctgacggctttttttc
ctgttccagcgccagcgcggccatatgctggctgatatctgccacgcgttcgataaaggcgctggtttctgctcctgacgaggttttaattt
gcaggatccccgcaggcgcgccatcacgctgacgaatggtcgctgaccagctttgcgcattttgaatttctgcaccgtgggaagatga
cgcccagtgtatcggcatcccgttgcgcagtgcgaacagcgaaacatgcgattcgttgagtacagattcgatgttacgacaaatgatttc
ccccatttcatgaaatggcgggctgctgcacatggcggcgagaatattgccttcaagctggcgaatctgccgttcttcggtgatatccga
gaaagtcattaccaggttctgcagatgcgcgagcacgtcataaaccgggctgatagaggctttaatccagatttttcaccggtgcgcgt
caacagcagaaattcgtcctgatcgcgggcggttttccatagcaactgttgtaaacgaatgcggttatcggcagggaattcaggaatgtt
caggagtgtatcgggctgcataccgctggcttcgctaatgcagtaaccaaacatttcggtaaatgcgcgattgcactgcaca

*trm*DF/R >1224 bp
acggtatcacccggacggaaggaaggtacgtcctgcttcatctgctcttgttcaagttgcttaataatgttgctcataatttaatctcttatcct
gggtaaactgatatctcgggggcttacgccatcccatcatgtttatgttgctgttgtgcgtgttccgttttgaactccgccagcaaccttgctt
gctcttcagtcagagccaggttttccagaagttcaggtcttctaagccaggtacggcccagcgactgtttcaaacgccagcgacgtatct
cggcatggttgcccgacagtaacactggcggaacttccatcccttctaacacctcaggccgcgtatagtgcgggcaatccagcaatcct
tcagcaaaggaatcttccgttgccgaggcttcatgtcccagtactcccggaataaaccgggaaacggagtcaatcagcgtcattgctgg
taactcaccaccactgagaacgtaatcgccgattgaccattcttcgtcaatttcggtttggatcacgcgctcatctataccttcgtagcgac
cgcacaccagaatcaatttttgattcgttgccagttcgctgacgcccgcttgatcaagcttgcgtccctgtggtgacagataaatcaccttt
gcgccttcacccgccgcggcttttgctgcatgaatggcgtcccgcaagggttgcaccatcattaacatccccggtccgccgccgtaag
gacgatcgtccacggtacggtgccggtcatgcgtgaagtcgcgaggactccagctctggatgctcagcaggccatttttaactgcccg
gccagttaccccgtaatcggtaattgcgcggaacatttcaggaaacaggctaattatgccaatccacatagcgccgtcttttaccgtttatc
cggtggtttaaaaaccaggatcccaatctacttcgattgaacgagtagtgagatcgactttcttgataacctgcccatcgaggaacggtac
gagacgttccttgataccaaacgcatctttcaggtttgccttaatgacgagaacgtcattagatccggtttccatcatatcgacgactttacc
gagatcgtagccttcagtggttactacctggcagcccatcaggtctttccagtagtagtcccccttcttcaagctgaggcagctgcgatga
atccacgacaatttcacaattcgtcagcaggttcgccgcat

Table S6. The sequences of RT-PCR products.

*ori*CqF/R>108 bp
accggttgatccaagcttcctgacagagttatccacagtagatcgcacgatctgtatacttatttgagtaaattaacccacgatcccagcca
ttcttctgccggatct

*que*AqF/R >109 bp
acaatgaccgcgcgccacggcgcactgtttgaagtcgaatttaatgatgaacgctcggtgctggatattctcaacagcatcggccatatg
ccgctgccgccgtatatcg

*ddp*AqF/R >105 bp
tgtgtcgccagagcaataggctcccagttcggatcgttatcggagtagagaaacgtcaggctggtgggtttgctcgtcactttatcccatt
cggctttggctttc

*cat*qF/R >111 bp
caacagtgccgttgatcgtgctatgatcgactgttatctctggcggtgttgacactggagcacctcaaaaacaccatcatacactaaatca
gtaagttggcagcatcaccc

*dos*PqF/R >108 bp
gtgcgaacagcgaaacatgcgattcgttgagtacagattcgatgttacgacaaatgatttcccccatttcatgaaatggcgggctgctgc
acatggcggcgagaatat

*trm*DF/R >115 bp
cccagcgactgtttcaaacgccagcgacgtatctcggcatggttgcccgacagtaacactggcggaacttccatcccttctaacacctc
aggccgcgtatagtgcgggcaatcca
